# The time course of visual foraging in the lifespan: Spatial scanning, organization search, and target processing

**DOI:** 10.3758/s13423-023-02345-8

**Published:** 2023-08-24

**Authors:** Marcos Bella-Fernández, Manuel Suero Suñé, Beatriz Gil-Gómez de Liaño

**Affiliations:** 1 Facultad de Psicología, Universidad Autónoma de Madrid, C/ Ivan Pavlov 6, 28029 Madrid, Spain; 2 Universidad Pontificia de Comillas, Madrid, Spain

**Keywords:** Foraging, Visual organization, Visual search, Lifespan

## Abstract

Visual foraging is a variant of visual search, consisting of searching for an undetermined number of targets among distractors (e.g., looking for various LEGO pieces in a box). Under non-exhaustive tasks, the observer scans the display, picking those targets needed, not necessarily all of them, before leaving the search. To understand how the organization of such natural foraging tasks works, several measures of spatial scanning and organization have been proposed in the exhaustive foraging literature: best-r, intertarget distances, PAO, and target intersections. In the present study, we apply these measures and new Bayesian indexes to determine how the time course of visual foraging is organized in a dynamic non-exhaustive paradigm. In a large sample of observers (279 participants, 4–25 years old), we compare feature and conjunction foraging and explore how factors like set size and time course, not previously tested in exhaustive foraging, might affect search organization in non-exhaustive dynamic tasks. The results replicate previous findings showing younger observers’ searching being less organized, feature conditions being more organized than conjunction conditions, and organization leading to a more effective search. Interestingly, observers tend to be less organized as set size increases, and search is less organized within a patch as it advances in time: Search organization decreases when search termination is coming, suggesting organization measures as potential clues to understand quitting rules in search. Our results highlight the importance of studying search organization in foraging as a critical source of understanding complex cognitive processes in visual search.

**Supplementary information:**

The online version contains supplementary material available at 10.3758/s13423-023-02345-8.

## Introduction

Imagine a soccer player controlling the ball in the middle of the field. The player must scan the field for teammates to pass the ball while avoiding rivals. Unfortunately for our player, the teammates are moving around, trying to find suitable positions to receive the ball, while rivals are trying to intercept the ball. Additionally, our player cannot spend more than a few seconds to find well-positioned teammates to pass the ball, or a rival may come to steal it. Now, imagine a security guard patrolling an area looking for suspicious activity. The area is crowded, and our guard must detect potential suspicious individuals (targets) among the crowd (distractors). The crowd is in constant movement, and potential targets and distractors are regularly coming in and out of the search area. At some point, the guard should consider that the area has been inspected enough and move to the following one.

These two tasks have some aspects in common. An observer must search for targets among distractors in movement in an adjustable environment. The number of potential targets (and distractors) is not always available. Ultimately, there are no constraints for the observer to determine when it is time to stop searching, and he/she must decide to determine the best moment to stop the search, pass the ball, or move on to continue the search. Under these circumstances, how is the search organized during the task to find targets in a dynamic and unpredictable environment? How does the search organization evolve during the search? How might the time course of the search determine the best moment to leave the search? Interestingly, how might this organization predict the moment to make the optimal decision to quit the search? And how does search organization change during different developmental stages? These are some of the questions we will try to address in the present work, in which we will study the time course of search organization using a video-game-like foraging dynamic task.

Visual foraging is increasingly used to understand human cognition in experimental psychology, largely inspired by animal foraging literature (for a review, see Bella-Fernández et al., 2022). In some paradigms (e.g., Kristjánsson et al., 2019, 2020), the participants are forced to collect every target in the display before switching to the next one (exhaustive foraging). In other paradigms (e.g., Gil-Gómez de Liaño & Wolfe, 2022; Wolfe, 2013; Wolfe et al., 2016, 2019) the observer is free to abandon the current display to start searching on others, usually less depleted (non-exhaustive foraging). Also, some studies have used static items (e.g., Kristjánsson et al., 2019, 2020; Ólafsdóttir et al., 2021), while others have used dynamic-in-movement foraging paradigms (e.g., Gil-Gómez de Liaño et al., 2022; Kristjánsson et al., 2022; Wolfe et al., 2016, 2019), mirroring our real-world examples with the soccer player or the security guard. Since non-exhaustive and dynamic tasks seem common and natural in many human activities, and less research is devoted to them in cognitive psychology, we will focus our study on those types of tasks to provide more scientific evidence in such dynamic non-exhaustive environments.

In foraging, observers’ visual scan-paths go from one target to another until the forager decides it is time to leave the display, seemingly when targets are scarce enough to make it optimal to search in richer patches (Charnov, 1976). At the beginning of every trial, the forager is presumed to attend to the most salient target/s and continue foraging the surrounding ones (Gil-Gómez de Liaño & Wolfe, 2022). Eye movements tend to optimize information processing (Hoppe & Rothkopf, 2019; Najemnik & Geisler, 2005, 2008). Thus, those targets within the functional visual field (FVF; Wu & Wolfe, 2022) would be the most detectable. That way, our visuospatial attention potentially follows an organized search (Smith & De Lillo, 2022; Woods et al., 2013), exploring and exploiting different areas within the same patch (Bella-Fernández et al., 2022). However, as the targets become scarcer as the search progresses and the forager needs more time to find other targets (remember that distractors are still there), there is a critical moment in which the forager must decide to move on.

Therefore, understanding search organization seems critical during visual search, as it directly relates to search efficiency (Clarke, Irons, et al., 2022c; Smith & De Lillo, 2022) and potentially to determining when it is the best moment to leave the search. As the organization seems to involve several aspects within search, like perceptual and attention processes comprising spatial movements to search from one region to another and decisions related to termination rules in search (Gil-Gómez de Liaño et al., 2022), several measures have been described to understand search organization: Best-r, intertarget distances (ITD), percentage above optimal scan-path (PAO), and the number of intersections between intertarget trajectories are the most common measures (see Methods for a more extensive description). Mark et al. (2004) described these indexes, which Woods et al. (2013) and Ólafsdóttir et al. (2021) later applied in exhaustive cancellation and visual foraging tasks with children. They found differences in those indexes between children and adults in visual search tasks for feature and conjunction conditions.

Another recent model (Clarke, Hunt, & Hughes, 2022a) captures spatial organization and foraging strategies in a complementary manner from a Bayesian perspective. Clarke et al. (2022a) proposed a family of models with parameters capturing trends to pick up targets in runs or switching between types of targets, preferences for particular target types, and spatial and proximity biases (see Methods section for a more detailed description). Unlike raw number of runs and switches, these parameters are independent of the number of trials, which makes them very appropriate in foraging paradigms like ours, with a varying number of targets collected from one trial to another. Parameters like those described by Clarke et al. (2022a), capturing bias toward picking the nearest targets and persevering in one direction are particularly interesting for us. Thus, in the present study, we will use both organization indicators and spatial parameters based on these Bayesian models to understand foraging organization in non-exhaustive and dynamic foraging tasks in the lifespan. We expect to find our organization measures and Bayesian indexes can help us to better understand dynamic foraging in non-exhaustive environments—from our knowledge, not previously tested in the literature. We also expect to find high correlations among most of the organization measures and the Bayesian parameters related to proximity and direction perseverance. Particularly, we expect significant correlations between best-r and angle perseverance from Bayesian models, while for proximity bias we expect higher correlations with mean ITD, PAO, and intersection rate (IR) indicators.

Our objective is not only to replicate previous findings in search organization for exhaustive tasks in our non-exhaustive dynamic tasks. Also, we aim to determine if organization rules similarly apply in non-exhaustive foraging, in which the elements are in constant movement (like in our real-world examples). Following previous studies in the field (e.g., Gil-Gómez de Liaño et al., 2022; Wolfe, 2013), we have developed a task in which observers are free to move on to following displays looking for targets in a dynamic and variable environment (that is, with in-movement-items under a variable number of elements—the set size—in each display). Potentially, a different search organization might arise as the observer does not need to pick every target within a display, and all the elements (targets and distractors) are in pseudo-random movement with constant speed. This new situation might require a continuous updating/adaptation of organization strategies, evolving differently as time goes by within each display/patch.

Another critical manipulation that differs from the previous studies in exhaustive foraging is set size. As set size (or item density) increases, FVF becomes smaller (Motter & Simoni, 2008). Larger FVFs are related to more efficient searches (Chan & So, 2007; Ebner et al., 2017); conversely, smaller FVFs are related to lower efficiency. In turn, as mentioned above, search efficiency is related to search organization (Clarke, Irons, et al., 2022c; Smith & De Lillo, 2022). Thus, we can hypothesize that larger set sizes may be related to poorer search organization (and thus poorer efficiency), especially for younger observers under conjunction conditions (Gil-Gómez de Liaño et al., 2022; Gil-Gómez de Liaño & Wolfe, 2022). Regarding the time course of the organization during a trial, easier, more salient targets tend to be the first ones picked (Wolfe, 2013), which means that the remaining targets tend to be scarcer and harder to find (larger item/target ratio or effective set size; see Gil-Gómez de Liaño & Wolfe, 2022; Wolfe et al., 2019). Thus, we hypothesize that as time goes by within a patch, the organization is lower (with remaining targets being harder to find), potentially harder for conjunction conditions and younger observers (Gil-Gómez de Liaño et al., 2022; Gil-Gómez de Liaño & Wolfe, 2022). Finally, the search difficulty associated with larger set sizes might have more impact on younger observers. We also expect that these difficulties will have accumulative effects with the difficulty associated with the condition (feature tasks being easier than conjunction tasks).

## Methods

### Participants

The sample used for the present study is the same as that used in Gil-Gómez de Liaño et al. (2022). Although all details of participants and methods are described in Gil-Gómez de Liaño et al. (2022), we will give the most relevant information for the reader to have a general and thorough idea of the methodology followed. Thus, the sample comprises 279 observers, ranging in age from 4 to 25 years from Madrid’s elementary, middle, and high schools. We maintained a minimum number of observers (21) at every age (4, 5, 6, etc.) to have enough power and to avoid underrepresentation of any age group. All participants had normal or corrected-to-normal vision. Parents or guardians gave written informed consent for each participant, and participants gave verbal or written consent. None of the observers had neurological or sensorial damage, motor impairments, or a diagnosis of schizophrenia or generalized developmental disorder (see Gil-Gómez de Liaño et al., 2022, for details).

### Equipment and stimuli

The experiments were programmed in MATLAB 7.10 using the Psychophysics Toolbox (Version 3; Brainard, 1997; Kleiner et al., 2007; Pelli, 1997). Stimuli were presented on a Microsoft Surface Pro i5, where observers responded by touching the screen. The monitor resolution was 1,400 × 1,050 pixels. As in Gil-Gómez de Liaño et al. (2022), stimuli were squares for the feature condition (green, blue, yellow, and red) and squares and circles for the conjunction condition (green and blue). The items were moving in random directions to make systematic searches more difficult. The item movement speed was constant at 44 pixels/s and changed directions at pseudo-random intervals.

Every display contained 60, 100, 140, or 180 items, with a proportion of targets randomly generated between 20% and 30% for each trial. The proportion of the target–distractor colors and shapes (in the conjunction condition) were randomized to avoid an excessive homogeneity across trials. Set sizes were also randomized across trials for each participant. Items appeared in random locations across the display, and starting locations and movement directions changed from trial to trial.

### Procedure

**Fig. 1:**
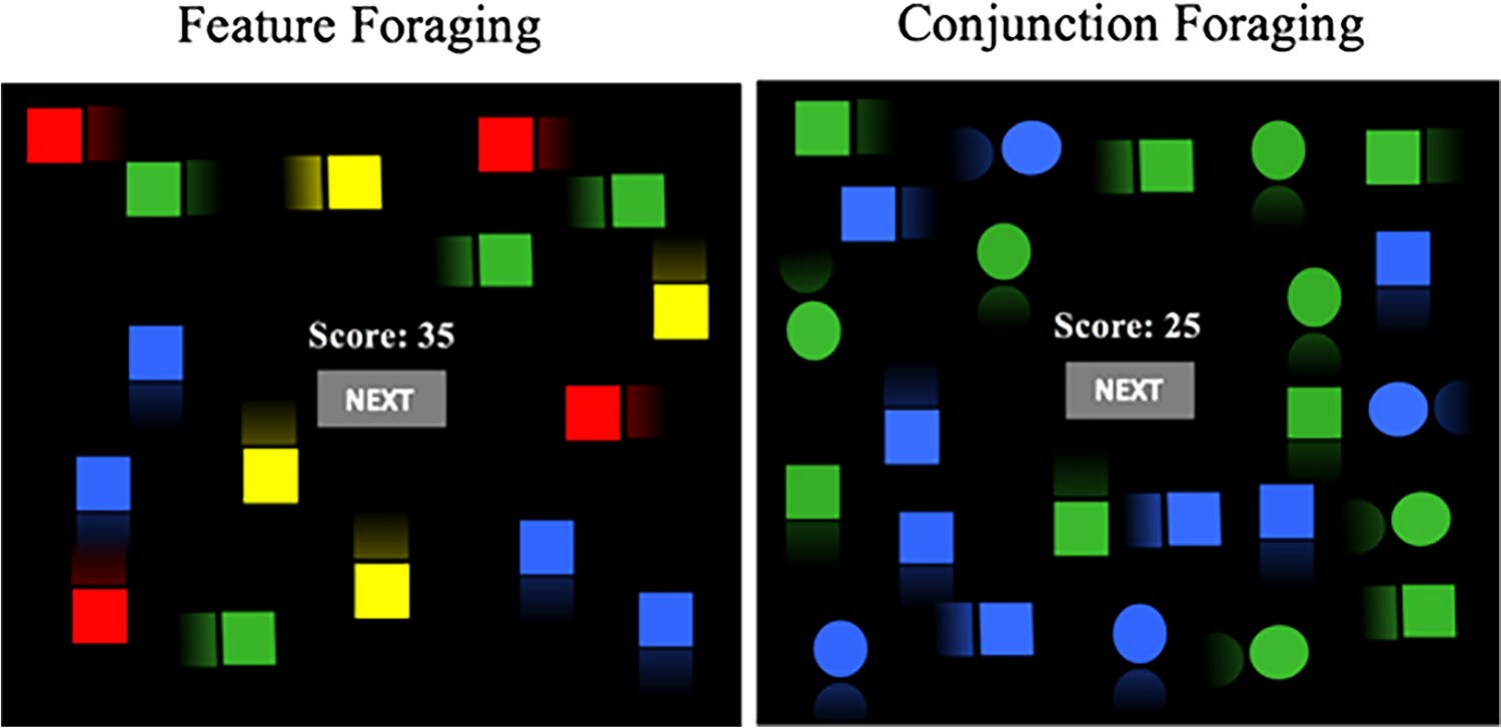
Example of Feature and Conjunction foraging tasks. For Feature tasks, targets and distractors are only distinguishable for one feature (color). For the Conjunction condition, targets and distractors are distinguishable for two features (color and shape). Taken with permission from Gil-Gómez de Liaño et al. (2022). (Color figure online)

Every observer must obtain 200 points by picking targets for each feature and conjunction condition (thus, 400 points for the whole task) by tapping them on a touchable tablet/computer. Each target was rewarded with 2 points, and every distractor cost 1 point. The score was visible on the screen. Tapping a target made it disappear from the screen. Tapping a distractor made a red cross temporarily appear on the distractor, but the distractor remained immediately after on the screen. The participants were instructed to find as many targets as possible, and they were free to leave a display/patch to start another one every time (by clicking on the “next” button on the center of the screen; see Fig. 1). A constant time gap (“traveling cost”) of 2 seconds went by between displays. Feature and conjunction conditions were counterbalanced, with half of the observers running first the feature condition and the other half running first the conjunction condition. All participants had a previous practice phase of 50 points to reach in every feature and conjunction condition. The complete task lasted between 20 and 40 minutes.

### Data analysis

All the analyses were performed in R, using the package “ForagingOrg” (Bella-Fernández, 2022) to estimate the foraging organization indicators. The packages “PairViz” (Hurley & Olford, 2022) and “TSP” (Hahsler & Hornik, 2007) were also used to calculate the shortest Hamiltonian paths, as described in the following sections. The package “rstan” (Stan Development Team, 2023) was used to estimate the parameter of the Bayesian foraging model from Clarke et al. (2022a).

#### Organization indicators

Organization measurements were calculated for every trial, defined as every patch visited with a variable number of targets collected. In our paradigm, because the observers are free to leave a trial/patch at any moment, every participant can visit a variable number of trials/patches^1^ and does not necessarily generate the same number of targets in each one, as it is variable within and between participants, since they can leave every patch at will.

##### Best-r

**Fig. 2:**
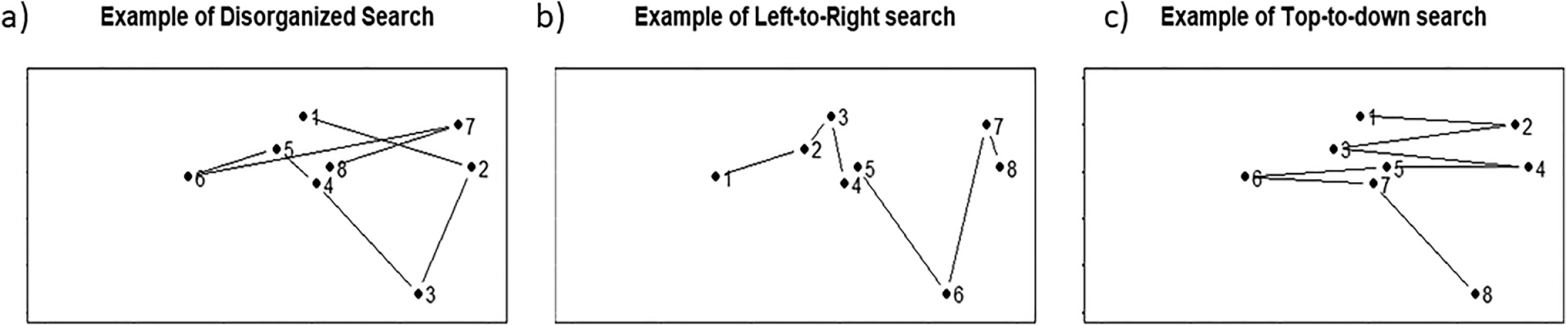
Graphical representation of the three examples exposed to explain how best-r works

In a foraging task, each target is located at a certain point in the display, which can be characterized with coordinates *x* and *y*. For every target *i,* it is possible to obtain its coordinates, *x*_*i*_ and *y*_*i*_. Then, we would have three vectors: the order in which the target has been collected: 1, 2, 3…; the *x* coordinates: *x*_*1*_*, x*_*2*_*, x*_*3...*_*,* and the *y* coordinates:* y*_*1*_*, y*_*2*_*, y*_*3....*_ Two Pearson correlations are then calculated: the correlation between the target order and *x* coordinates and the correlation between the target order and *y* coordinates. The best-r is the greatest of those two correlations (in absolute value). Best-r is good at detecting reading- or scanner-like searching patterns and, more generally, patterns with strong horizontal or vertical components, but not for other types of searches, like spiral-searches. Figure 2 shows simulated examples of three different searches with different calculated best-r indexes according to the correlation procedure. For the random/disorganized simulation (a), the calculated best-r index is 0.255, while for organized, scanner-like left-right (b) and up-down (c) searches, the best-r are 0.970 and 0.967, respectively (both close to the maximum value 1). A larger best-r indicates more organized foraging.

Best-r has shown to increase with age in exhaustive and static foraging (Ólafsdóttir et al., 2021; Woods et al., 2013). Also, best-r was generally larger in feature than in conjunction foraging exhaustive and static tasks (Jóhannesson et al., 2016; Kristjánsson et al., 2022; Ólafsdóttir et al., 2021; Woods et al., 2013). No evidence of interaction between age and condition was found (Ólafsdóttir et al., 2021). Also, Woods et al. (2013) found that best-r was related to search accuracy only in conjunction search, not in feature. Finally, Ólafsdóttir et al. (2021) found a positive correlation between best-r and the number of switches between types of targets, shown in the next section.

##### Mean intertarget distance (ITD)

**Fig. 3:**
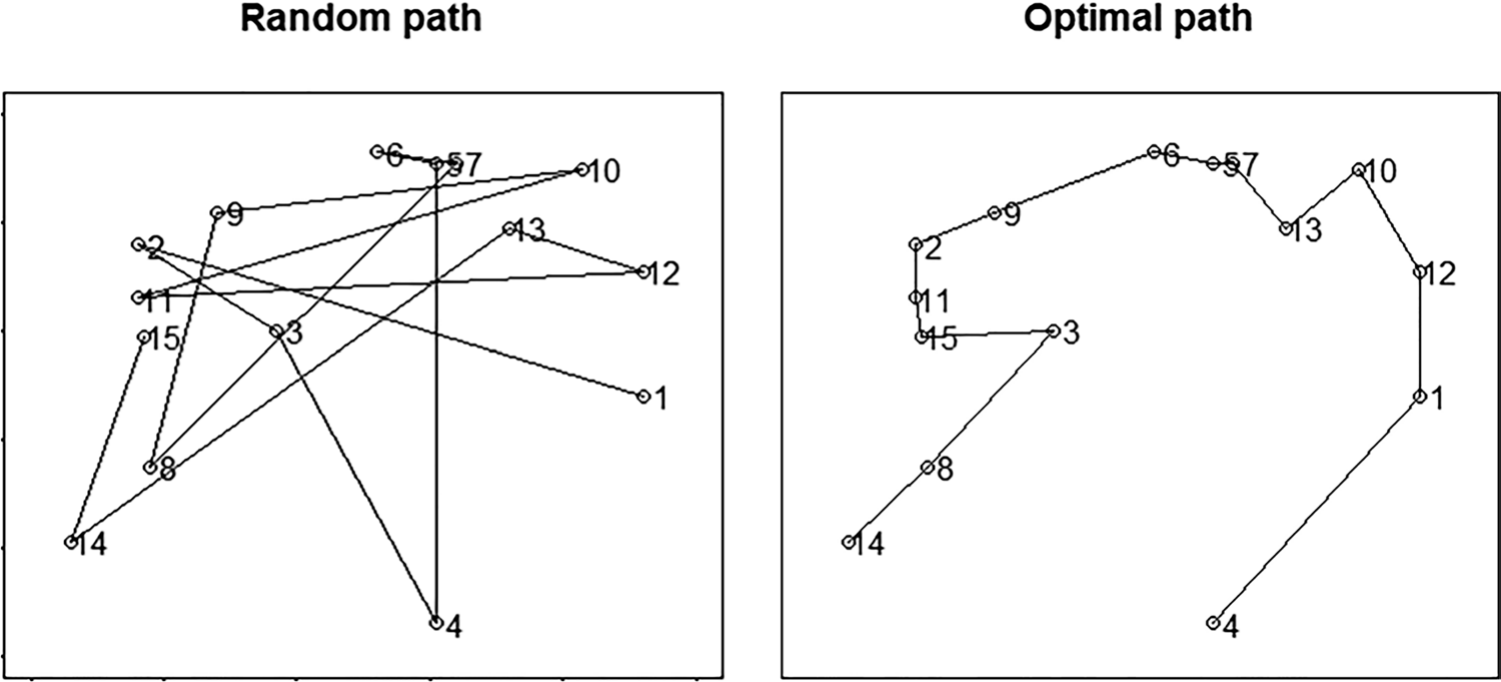
Random and optimal path. The mean ITD and the PAO capture the optimal paths, although in different scales

The mean ITD is the mean of the Euclidean distances between consecutive targets. It is usually used a measure of search organization. Larger mean ITDs mean more complicated and less organized searches (see Fig. 3).

Because the number of targets and target density might influence the calculation of the mean ITD, standardized measures have been proposed (e.g., Dalmaijer et al., 2015). These standardizations are based on the relative distances between all the targets. Computing mean ITD considering different instants in every patch, since our dynamic task makes ITDs fluctuate at every moment, could not result as efficient as classic static mean ITD calculations.^2^ However, we present mean ITD results, because they show similar and coherent results compared with the rest of the indicators and previous works in the field, as we will show in the Results section. Previous works in exhaustive foraging show that the mean ITD decreases as age increases in exhaustive searches (Ólafsdóttir et al., 2021; Woods et al., 2013), being this decrease only ameliorated from age 17 (Woods et al., 2013). For all ages, mean ITD is lower for feature foraging than for conjunction foraging, without a significant interaction between age and condition (Kristjánsson et al., 2022; Ólafsdóttir et al., 2021). Unlike best-r, mean ITD is not related to accuracy for any foraging condition (Woods et al., 2013). So, computing mean ITD in dynamic environments could not be as inefficient as expected, even considering ITD fluctuations.

##### PAO

For every set of targets, there are many ways to draw a path passing through each target location once and only once. Mathematically, each one of these paths is named a Hamiltonian path (e.g., Wilson, 1972). From a set of Hamiltonian paths, one is optimal; that is, the shortest path length. The actual scan-path is another (presumably suboptimal) Hamiltonian path, whose length can be compared with the optimal one. This comparison can be expressed as a percentage; for instance, a percentage of 50% means that the actual path is 1.5 times as large as the optimal path.

Equation 1 is the formula to find the PAO, given the optimal and the actual path lengths. The larger the PAO, the less organized the search. For instance, a PAO of 10% indicates a slightly larger path than the optimal path. Thus, that path would be a quite optimal path. A PAO of 100% means that the actual path length is double the optimal path length. Thus, a worse option than the 10% previous one in optimality terms.

1 $$PAO=\left(\frac{Actual\; scanpath\; length}{Optimal\; scanpath\; length}-1\right)*100.$$

Finding the shortest Hamiltonian path is an np-hard problem,^3^ and its solution is based on heuristics. When humans face straightforward presentations of the traveling Salesman Problem (TSP), they find close-to-optimal solutions (McGregor & Chu, 2011; Tenbrink & Wiener, 2009). Similarly, applied to foraging, Ólafsdóttir et al. (2021) found that PAO decreases as age increases and that PAO was larger for conjunction than for feature foraging. However, dynamic environments with moving targets, such as our paradigm, may not represent straightforward presentations of the TSP. PAO values might be larger than those found in other foraging paradigms in static environments.

However, the PAO, like the rest of the indicators, is calculated a posteriori in the present work, considering the *x* and *y* coordinates of the targets at the moment they were collected. In a dynamic environment, as the targets are moving, the optimal path may constantly change, so the observer may have new-updated information every time a target is picked to recalculate the optimal path. This might be a limitation for PAO in our dynamic environment. However, using the PAO with the actual *x-y* coordinates at every target picked may give an approximation of the PAO value in our task worth to study. Actually, the rate of movement is not fast enough to generate extremely different values, so it could be a good measure even under dynamicity. Indeed, as we will see below, the correlation of PAO with the rest of the measures is moderate to high, so the approximation we have used seems to work well. Also, the PAO results replicate essential previous effects found in exhaustive and static foraging (Ólafsdóttir et al., 2021). Thus, using PAO as an organization measure can be a good index, even under dynamic conditions.

##### Intersection rate (IR)

Another organization measure widely used in the literature is the number of intersections between nonconsecutive targets in the scan-path. To count the number of intersections, we used an algorithm similar to the one described in Donnelly et al. (1999). For two displays with the same number of targets collected, a larger number of intersections indicates a lower degree of organization. In paradigms like ours, the number of targets collected varies from one trial to another, and this variability can affect the number of intersections: the larger the number of targets, the larger the number of intersections. To control this, the intersection *rate* (IR; Mark et al., 2004) was calculated instead. The intersection *rate* consists on dividing the number of intersections between the number of targets collected.

##### Spatial and strategy biases indexes from Bayesian models

As described above, Clarke et al. (2022a) proposed a Bayesian model based on sampling without replacement to estimate some strategy and spatial biases in target selection. The simplest version of this model, named “bag foraging model,” is based on sampling without replacement estimating the probability of picking the same target-type as the last one collected, p_s_. The model adds a second parameter, p_a_, which estimates the preference for a particular target type. These two parameters do not seem directly related to spatial organization measures, but with the object-based attentional set imposed during the task. Thus, higher values of any of these parameters would lead to larger runs (picking the same target over and over again), but for different reasons. In a more complicated version of the model, two parameters regarding spatial biases (and more interesting for us for the present study purposes) are added. The first of these parameters, ρ_d_, captures a bias toward collecting the targets that are closer to the currently picked one. Another parameter, ρ_Θ_, captures the “perseverance” or the trend to keep searching in the same direction, not varying the saccade angles. See Clarke et al. (2022a, b) for a deeper description of these parameters and their mathematical foundation.

In sum, larger best-r values (ranging between 0 and 1) indicate greater organization. The other indicators (mean ITD, PAO, and IR) show greater/better organization under lower values and can be any positive number from zero to infinite. p_s_ values above 0.5 (ranging from 0 to 1) indicate a trend to make large runs and, conversely, lower values indicate a trend to switch between targets. Larger p_s_ values (also ranging from 0 to 1) indicate a trend to pick up targets of a certain type. Larger ρ_d_ values indicate a trend to pick up closer targets. Last, shorter ρ_Θ_ values indicate a trend to change the path direction and larger ρ_Θ_ values indicate a trend to keep the direction constant. ρ_Θ_ may have a positive or negative sign, depending on the side (right or left) of the direction bias. In this context, we only interpret the absolute value of ρ_Θ_, denoted as |ρΘ |, regardless its positive or negative sign, because we are interested on the direction bias irrespective of the side.

### Linear mixed-effects models

Linear mixed models (LMM) were fitted for each measure using the R package “lme4” (Bates et al., 2015; see also Brown, 2021). For every measure, we estimated a linear mixed-effects model with four fixed-effect factors: age, condition (feature and conjunction), set size (60, 100, 140, and 180), and bin (two bins: bin1 and bin2). To analyze the time course of foraging organization, we divided each trial into two halves or bins, each one containing data from the first and the second half of the targets collected within that trial, respectively.^4^ It is important to note that available evidence in previous exhaustive foraging tasks suggests that age could not follow a strictly linear function in search organization (e.g., Ólafsdóttir et al., 2021; Woods et al., 2013). It is also supported for other cognitive functions in developmental studies that age follows a function in which changes are more pronounced from earlier childhood ages 5–7, and from a certain age (8–10), changes are smoother, generating more logarithmic-like functions (e.g., Anderson, 2002; Gil-Gómez de Liaño et al., 2020). Thus, instead of using the age as a linear predictor, we used the natural logarithm of the age to fit it to a logarithmic model, as it seems more accurate to previous results. We adjusted this log function only for age; the rest of the factors fit linear models. Set size has shown to be essentially linear in lots of studies in visual search and foraging (e.g., Wolfe, 2021a), while dichotomous factors “condition” and “bin” should fit well in linear models as well (see Ólafsdóttir et al., 2021 for feature/conjunction results in foraging exhaustive tasks).

### Model comparison

Besides the LMM analyses, and following Burnham et al. (2011), we contemplated all theoretically plausible models to compare them using Akaike information criterion (AIC) measures. Instead of estimating a signification level for every comparison, Burnham et al. (2011) described a method based on AIC for every plausible model. For plausible models, we mean those models suggested by the theory and our hypotheses (see the Introduction); otherwise, we should test an unmanageable number of models (see Table 1 for a summary of the models tested). Among these plausible models, the one with the lower AIC was chosen as the best approximation. The rest of the models are compared with the “best” model through the Δ_i_ indicator, which is simply the difference between the AICs for a particular model and for the best one. (For the interested reader, the procedure is described in detail in Annex 1.)

**Table 1:** Description of the 10 plausible models

Model	Effects	Number of parameters
Model 1	Age + Condition + Set Size + Bin	7
Model 2	Model 1 + Age × Set Size	8
Model 3	Model 1 + Condition × Set Size	8
Model 4	Model 1 + Age × Set Size + Condition × Set Size	9
Model 5	Model 1 + Age × Bin	8
Model 6	Model 1 + Bin × Condition	8
Model 7	Model 1 + Age × Bin + Condition × Bin	9
Model 8	Model 1 + Age × Set Size + Condition × Set Size + Age × Bin + Bin × Condition	11
Model 9	Model 8 + Age × Set Size × Condition	12
Model 10	Model 8 + Age × Set Size × Condition × Bin	13

## Results

When testing the model fitting for all the organization indicators, we found that the best-fitting model for all of them, in all cases, was Model 10; that is, the model including the four-way interaction as significant (see Table 1). We can see the results of those model fits in Table 2 for every organization indicator tested.

**Table 2:** Results of the fitting model process for best-r, PAO, intersection rate, and ρ_d_

	Best-r			Mean ITD			PAO			Intersection rate			ρ_d_		
Model	AIC	AIC_c_	Δ_i_	AIC	AIC_c_	Δ_i_	AIC	AIC_c_	Δ_i_	AIC	AIC_c_	Δ_i_	AIC	AIC_c_	Δ_i_
Model 1	−646.9	−646.5	28.9	78279	78279.4	103.2	68160	68160.4	73.2	−218.9	−218.4	77.8	19063	19063.4	39.0
Model 2	−655.2	−654.7	20.7	78274	78274.5	98.4	68162	68162.5	75.4	−217.6	−217.1	79.2	19056	19056.5	32.2
Model 3	−657.0	−656.5	18.9	78268	78268.5	92.4	68159	68159.5	72.4	−217.5	−216.9	79.4	19058	19058.5	34.2
Model 4	−664.9	−664.2	11.1	78263	78263.7	87.5	68161	68161.7	74.5	−216.2	−215.6	80.7	19053	19053.7	29.3
Model 5	−645.7	−645.1	30.2	78327	78327.5	151.4	68149	68149.5	62.4	−218.9	−218.4	77.9	19054	19054.5	30.2
Model 6	−654.8	−654.3	21.1	78201	78201.5	25.4	68115	68115.5	28.4	−226.7	−226.1	70.2	19062	19062.5	38.2
Model 7	−653.8	−653.2	22.2	78197	78197.7	21.5	68106	68106.7	19.5	−226.3	−225.7	70.6	19054	19054.7	30.3
Model 8	−671.9	−670.9	4.4	78181	78182.0	5.8	68107	68108.0	20.8	−223.7	−222.7	73.5	19044	19045.0	20.6
Model 9	−676.1	−675.0	0.4	78182	78183.2	7.0	68109	68110.2	23.0	−226.6	−225.4	70.8	19024	19025.2	0.8
Model 10	**−676.5**	**−675.3**	**0.0**	**78175**	**78176.2**	**0.0**	**68086**	**68087.2**	**0.0**	**−297.4**	**−296.3**	**0.0**	**19023**	**19024.4**	**0.0**

For all the models, *n* = 279. In bold, the best-fitting model. AIC = Akaike information criterion. AIC_c_ = corrected Akaike information criterion. Δ_i_ = difference between the corrected Akaike information criterion of a certain model and the lowest corrected Akaike information criterion.

With Model 10 and the four-way interaction being significant for all organization indicators, as shown in Table 2, we show the results and figures to understand how those effects and interactions explain how the observers organize search in our foraging tasks. We expose the results considering each organization indicator separately. We plot 2D and 3D figures for all the selected indicators, to show the results in the best way to understand how observers organize search upon the four factors tested: time-bin (1 and 2), condition (feature/conjunction), Age (5–25) and set size (60,100,140, and 180).

### Best-r

**Fig. 4:**
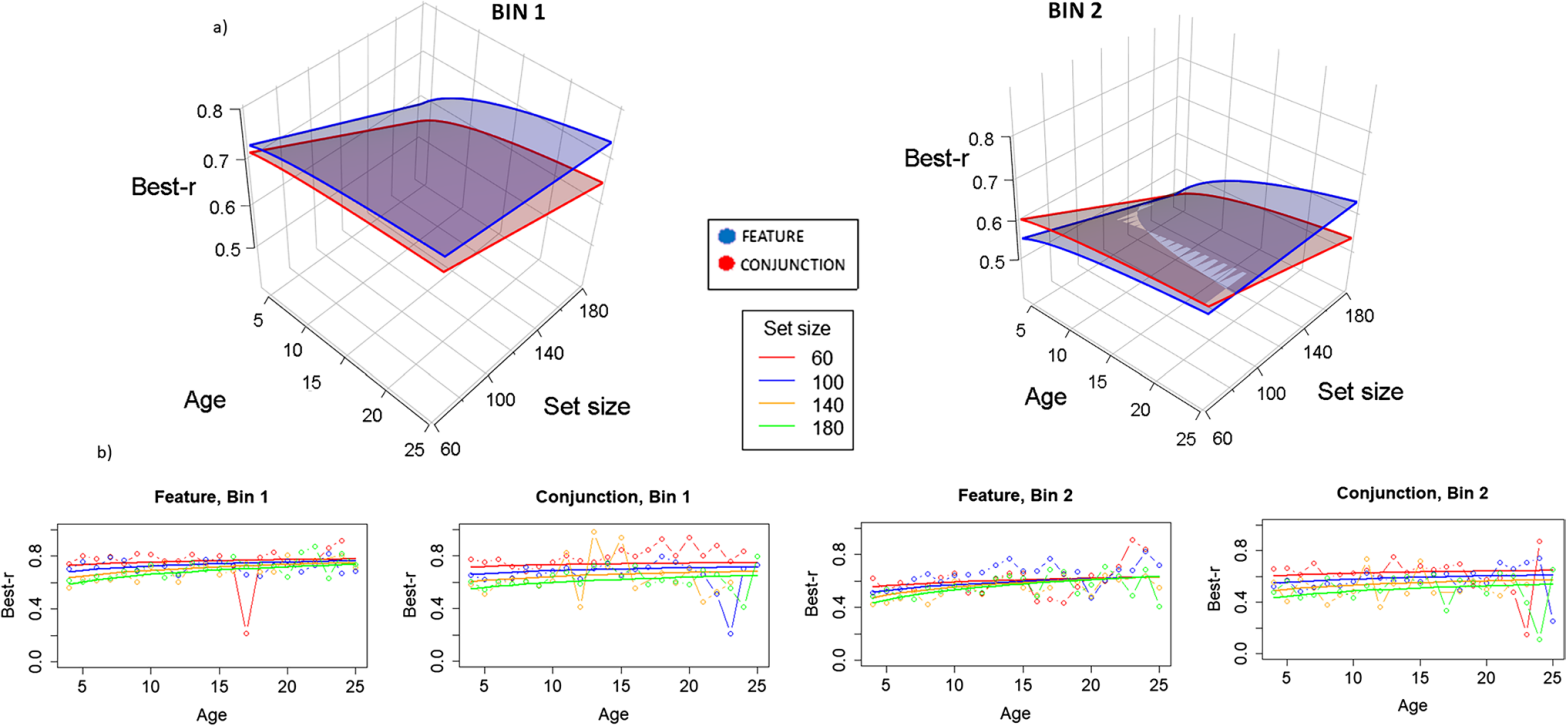
Model representations for best-r analysis. Remember that for best-r, the larger the value, the better the organization. **a)** 3D representation. Different graphs represent Bin 1 and Bin 2, showing that best-r diminishes as time advances to Bin 2, leading to worse organization. Different planes represent feature (blue) and conjunction (red) conditions, showing that depending on bin, organization differs, being better for feature in Bin 1, with larger values, but oscillating as time advances in Bin 2. Set size essentially shows lower best-r values as set size increases (so worse organization in this case), as shown by the slopes represented in the right lines of the planes; this effect is lower for older participants. Age is set to a logarithmic function showing essentially that age functions for shorter set sizes are flatter (in both bins; see the lines drawing the left side of the planes), while the right inside side clearly shows a logarithmic function (at larger Set Size 180 condition), with younger children showing lower best-r levels, so worse organization. **b)** 2D representation. Solid lines represent the fitted model and lines with dots represent the marginal means for the raw data. Left images are for Bin 1 (Feature and Conjunction), and right images for Bin 2 (again, Feature and Conjunction). By these 2D graphs we can better see the effects of age and set size in the intermediate levels of the factors, showing better organization as set size decreases for younger children, while the differences diminish and even disappear in feature conditions for older observers. (Color figure online)

In Fig. 4, we can see the plots of the linear regressions estimated by the model for best-r, which can help in understanding the interactions and effects of Model 10. Bin (Bin 1–Bin 2) is represented in the left/right 3D images and below in the 2D images. Condition is represented in different planes in the 3D graphs, and in the left/right images below the 3D graphs. Age and set size are represented in the axes in the 3D images, and set size in different lines for the 2D graphs. As we can see, best-r is larger in Bin 1 than in Bin 2, suggesting that organization tends to decrease in a trial. Best-r is also lower for larger set sizes (except for older participants) and becomes larger as age increases. Furthermore, best-r is slightly larger for feature than for conjunction. In feature, best-r tends to be less dependent of set size as age increases, but this effect is not significant in conjunction.

### Mean ITD

**Fig. 5:**
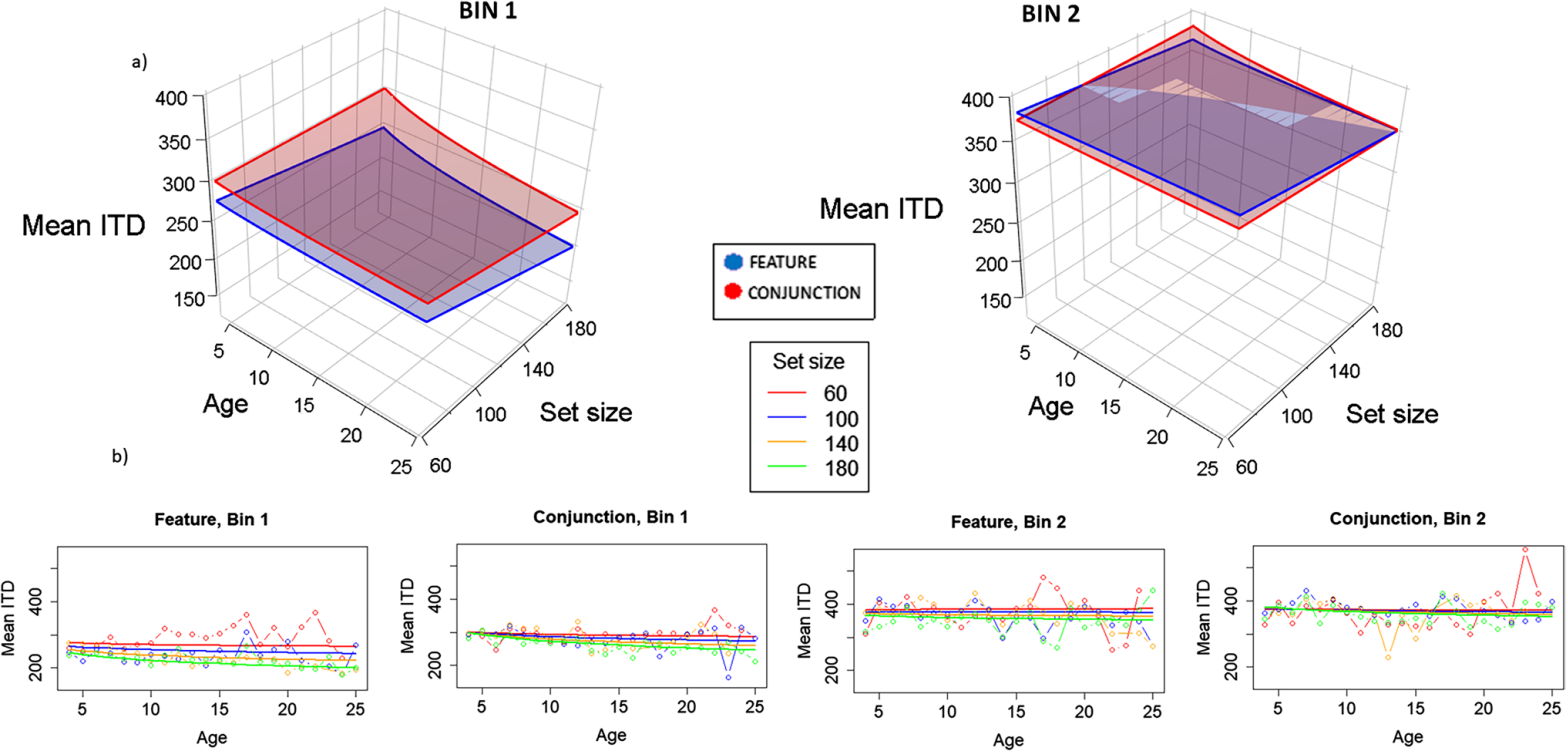
Model representations for mean ITD analysis. Remember that for mean ITD, the shorter the value, the better the organization. **a)** 3D representation. Different graphs represent Bin 1 and Bin 2, showing that the mean ITD increases as time advances to Bin 2, leading to worse organization. Different planes represent feature (blue) and conjunction (red) conditions, showing that depending on bin, organization differs, being better for feature in Bin 1, with shorter values, but oscillating as time advances in Bin 2. The effect of age in Bin 1 is slight, but clear in larger set sizes, showing that organization is better for larger ages; this effect disappears in Bin 2, showing essentially flat functions except for conjunction condition in the largest set size. Set size itself has an effect only in Bin 1 and only for older ages, being larger in feature than in conjunction condition. Regarding condition, in general, mean ITD is shorter (organization is better) for feature than for conjunction, except in Bin 2, with younger participant and larger set size, where the effect is the opposite. **b)** 2D representation. Solid lines represent the fitted model, and lines with dots represent the marginal means for the raw data. Left images are for Bin 1 (Feature and Conjunction), and right images are for Bin 2 (again, Feature and Conjunction). By these 2D graphs we can better see the effects of age and set size in the intermediate levels of the factors, showing better organization as set size increases and age increases (but only for Bin 1). (Color figure online)

In Fig. 5, we can see the 3D plots for the linear mixed regressions for mean ITDs as a function of all factors (again, Model 10 was the best-fitting one). Remember that for mean ITDs, the larger the index, the less organized the search (unlike for best-r). Mean ITDs slightly decrease with age. In Bin 1, the index is larger for conjunction than for feature, although this effect is mitigated in Bin 2. Mean ITD is also larger for Bin 2 than for Bin 1.

### PAO

**Fig. 6:**
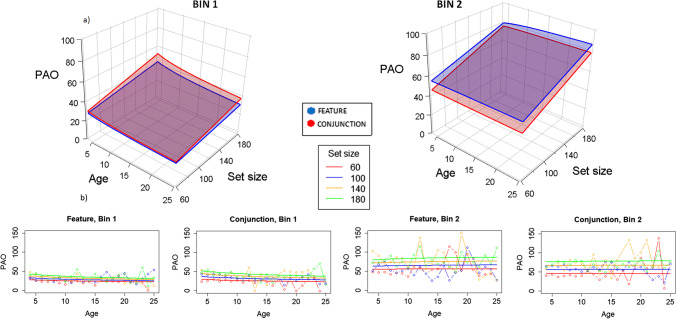
Model representations for PAO analysis. Remember that for PAO, the shorter the values, the better the organization. **a)** 3D representation. Different graphs represent Bin 1 and Bin 2, showing that PAO increases as time advances to Bin 2, leading to worse organization. Different planes represent feature (blue) and conjunction (red) conditions, showing that depending on bin, organization differs, being better for feature in Bin 1, with shorter values, and better for conjunction in Bin 2. Set size essentially shows larger PAO values as set size increases (so worse organization), as shown by the slopes represented in the right lines of the planes. Age is set to a logarithmic function showing essentially flat functions for age for shorter set sizes (in both bins; see the lines drawing the left side of the planes), while the right-inside side shows a smoothing logarithmic function (at larger Set Size 180 condition), with youngest children showing higher PAO levels, so worse organization. **b)** 2D representation. Solid lines represent the fitted model and lines with dots represent the marginal means for the raw data. Left images are for Bin 1 (Feature and Conjunction), and right images for Bin 2 (again, Feature and Conjunction). PAO is larger in Bin 2 than in Bin 1 (thus, organization decreases from Bin 1 to Bin 2) and in conjunction than in feature (organization is lower in conjunction). PAO is lower for lower set sizes than for larger set sizes (organization is better for lower set sizes), and this difference is amplified in Bin 2 compared with Bin 1. In Bin 1, but not in Bin 2, organization increases with age. (Color figure online)

Figure 6 shows the plots for the linear regressions in PAO, considering again Model 10. PAO is larger in Bin 2 than in Bin 1. In Bin 1, PAO is larger in conjunction than in feature, which suggests that organization is better in feature and at the first half of each trial; this trend is reversed in Bin 2. In Bin 1, PAO tends to increase with age, but this effect practically disappears in Bin 2. PAO is lower (and thus organization tends to be larger) for lower set sizes, and this effect is larger as the trial advances.

### Intersections rate (IR)

**Fig. 7:**
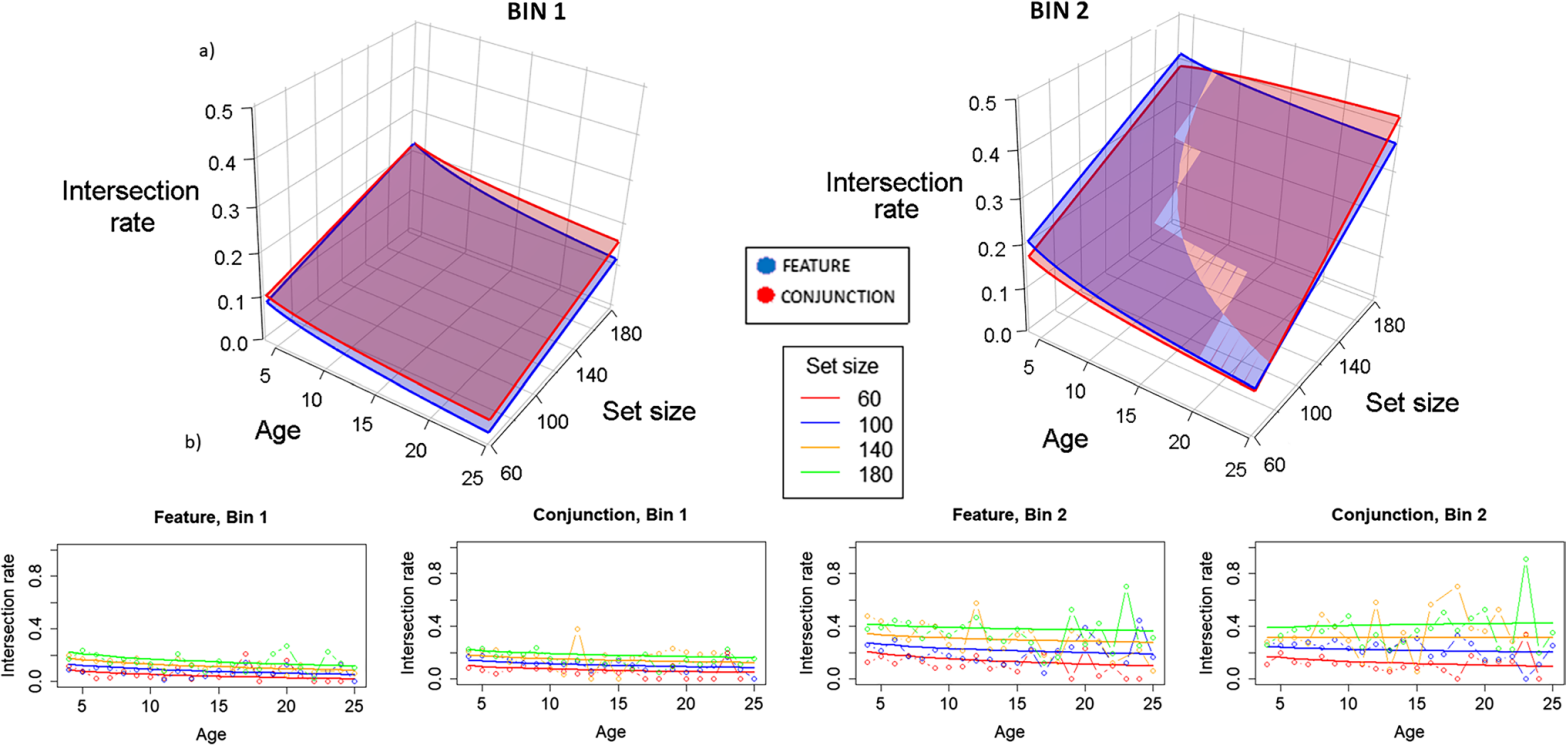
Model representation for IR analysis. Remember that for IR, the shorter the value, the better the organization. **a)** 3D representation. Different graphs represent Bin 1 and Bn 2, showing that IR increases as time advances to Bin 2, leading to worse organization. Different planes represent feature (blue) and conjunction (red) conditions, showing that depending on bin, organization differs, being better for feature in Bin 1, with shorter values, and oscillating for conjunction in Bin 2 upon both age and set size. Set size essentially shows larger IR values as set size increases (so worse organization), as shown by the slopes represented in the right lines of the planes. Age is set to a logarithmic function showing essentially larger values for youngest children (so, worse organization) in all conditions except in conjunction at Bin 2, for Set Size 180, at which the function looks rather flat, showing no differences as on age. **b)** 2D representation. Solid lines represent the fitted model, and lines with dots represent the marginal means for the raw data. Left images are for Bin 1 (Feature and Conjunction), and right images for Bin 2 (again, Feature and Conjunction). IR is larger for Bin 2 than for Bin 1, indicating that organization decreases over time inside a trial. IR is lower for lower set sizes, indicating that organization decreases as set size increases; this effect is more apparent in Bin 2 than in Bin 1 (the lines are more separated in Bin 2). In Bin 1, and only slightly in Bin 2, IR tends to decrease with age, indicating that organization increases with age. (Color figure online)

As shown in Fig. 7, the pattern of results is pretty like that found for PAO, except that for Bin 1, feature and conjunction are more similar and the increase of organization (decrease in IR) with age remains in Bin 2. Remember, that Model 10, considering the four-way interactions significant is the best fitting model for all indexes.

### Bayesian parameters

Table 3 shows the correlations between the organization indicators and the parameters from Clarke et al. (2022a). We can see that the first two parameters, p_a_ and p_s_, do not show significant correlations. As mentioned in the methods, those two parameters seem to be more related to strategy-search processes object-based (already studied in Gil-Gómez de Liaño et al., 2022) rather than spatially organization measures. However, the proximity parameter, ρ_d_, correlates moderately with best-r and strongly with the rest of the organization measures. Also, the absolute value of parameter ρ_Θ, |_ ρ_Θ|,_ shows small correlations with the organization measures. As seen in the Methods, these last parameters can be more related to aspects of spatial organization, as they consider the probability associated to pick a spatially-nearby target and the trend of observers to change the direction-path of a given trajectory while picking targets.

**Table 3:** Correlation matrix between organization measures and parameters from Clarke et al. (2022a)

	p_a_	p_s_	ρ_d_	|ρ_Θ_|
Best-r	0.014	−0.077	0.362	−0.081
	*p* = .6062	*p* = .00393	*p* < .0001	*p* = .0023
Mean ITD	−0.023	0.021	−0.749	−0.014
	*p* = .3773	*p* = .4246	*p* < .0001	*p* = .5786
PAO	−0.016	0.073	−0.705	0.079
	*p* = .5433	*p* = .006677	*p* < .0001	*p* = .0031
Intersection rate	−0.015	0.054	−0.629	0.052
	*p* = .5729	*p* = .04341	*p* < .0001	*p* = .0539

In the case of ρ_⊝_, we used the absolute value instead of the raw value because the sign of the direction bias is informative of the direction (left or right) of the bias, not its strength

**Fig. 8:**
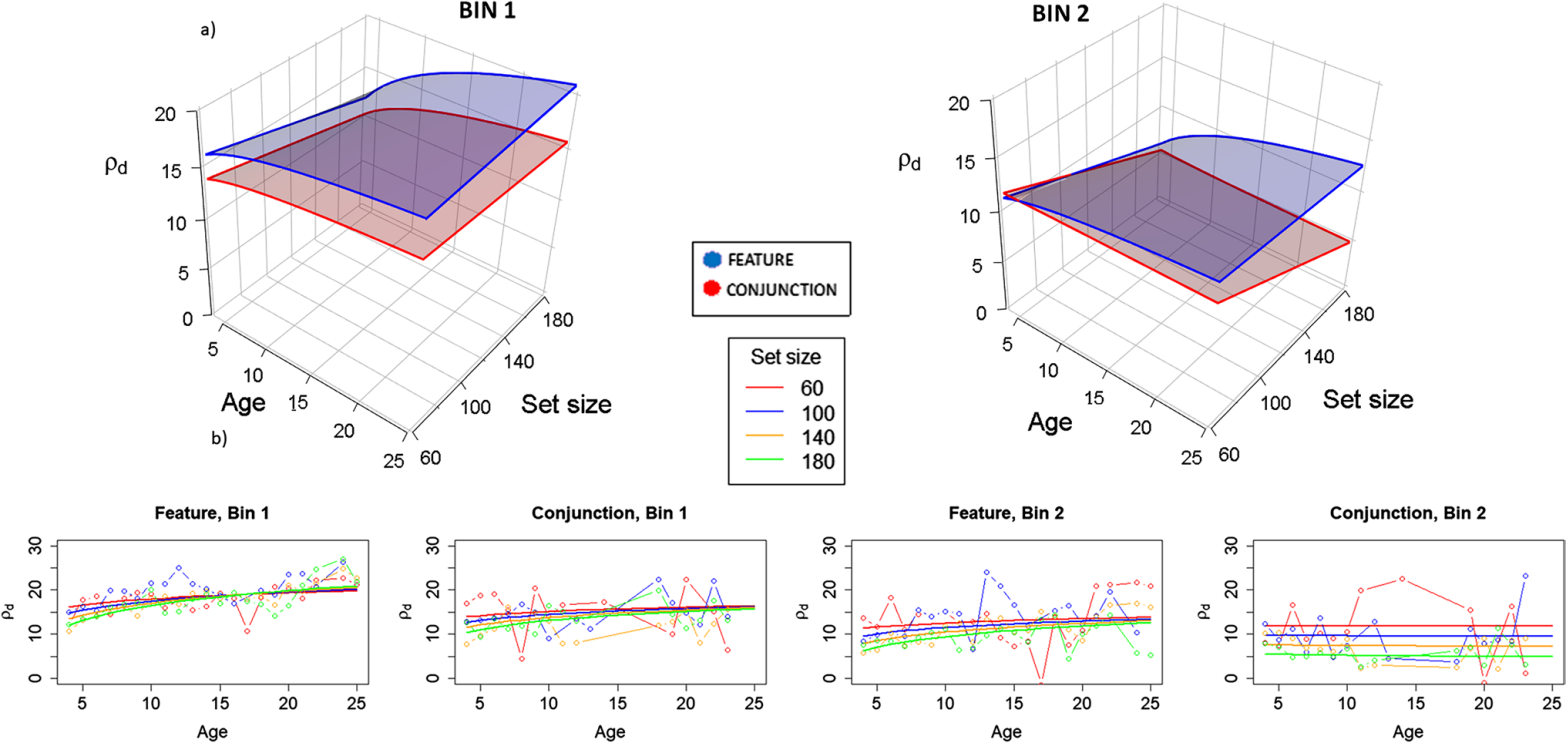
Model representation for ρ_d_ analysis. Remember that for ρ_d_, the larger the value, the better the organization. **a)** 3D representation. Different graphs represent Bin 1 and Bin 2, showing that ρ_d_ decreases as time advances to Bin 2, leading to picking further targets (which makes sense since targets are less frequent in Bin 2, the number of distractors being the same). Different planes represent Feature (blue) and Conjunction (red) conditions, showing that in general, the closer targets are better identified at Feature conditions, especially for older observers, as seen in the clear logarithmic function shown in all planes, but especially in the blue (feature) ones. Set size essentially shows flatter functions, showing a similar trend for all set size conditions, although they are a bit larger for feature at Bin 2. Age effects essentially show up at larger set sizes, seen un the curvature of the planes at 180 set size condition, especially for the feature (blue) conditions. **b)** 2D representation. Solid lines represent the fitted model and lines with dots represent the marginal means for the raw data. Left images are for Bin 1 (Feature and Conjunction), and right images for Bin 2 (again, Feature and Conjunction). ρ_d_ is larger for Bin 1 than for Bin 2, suggesting that organization decreases as time advances in a trial. On Bin 1, feature condition is more organized than conjunction, but this effect tends to decrease in Bin 2 in the conjunction condition. Except for conjunction in Bin 2, an effect of age is apparent, older participants being more organized than younger ones. Regarding set size, organization is larger for lower set sizes, but this effect disappears for older participants (again, Bin 2 in the conjunction condition is an exception, where this effect persists). (Color figure online)

Findings from Table 3 show that the parameter ρ_d_ might be a good organization indicator worth testing, strongly related with all the organization indicators except the best-r. Although statistically significant, correlation with |ρ_Θ_| are too small to be worth testing. Thus, we fitted mixed linear models with ρ_d_ as dependent variable. Figure 8 shows the fitted model for ρ_d_ (the probability associated to pick a spatially nearby target). The organization is better with age and tends to decline over the course of each trial. Organization is slightly better for feature than for conjunction, and it is also better for lower set sized compared with larger set sizes.

In summary, the best model fitting the data for all organization indexes is Model 10 (Table 1), considering all main effects of factors and the four-way interaction. For all indexes, again, organization decreases in the second half of every trial (patch) compared with the first half, showing that as the participant is reaching a decision criterium of quitting (“it is time to leave the search”), the search becomes more disorganized. Also, all indicators generally show that organization increases with age, although some interactions depending on set size and condition (feature/conjunction) arise for some indexes. For the set size effects, except for mean ITD and at some conditions for Best-r and ρ_d_, the organization tends to be larger with lower set sizes (60) than with larger set sizes (180). Lastly, in the first bins, that is, at the beginning of every search, the organization is larger in feature simpler conditions than in conjunction ones for all indexes. Still, this difference is less evident in the second bins, where more variability arises among different organization indicators.

## Discussion

Studying organization in non-exhaustive dynamic foraging in the lab by using a video game-like task mimicking more natural foraging in the real world (following our soccer player and security guard examples looking for teammates and threats in a crowd), we have essentially replicated previous results found in exhaustive foraging experiments (Kristjánsson et al., 2022; Ólafsdóttir et al., 2021; Woods et al., 2013). Indeed, our task added motion and new set size conditions compared to those used by Kristjánsson et al. (2022) or Ólafsdóttir et al. (2021); plus, no restrictions to target collection. This lack of restrictions left participants deciding when to quit the search, generating non-exhaustive foraging (e.g., Wolfe, 2013). Thus, it seems that similar organization rules apply in non-exhaustive and exhaustive foraging after all, although we will explain some nuances.

The replication of exhaustive foraging results gives us an answer to one of the critical questions we aimed to address in this study: how the search is organized during non-exhaustive foraging. The answer seems easy; non-exhaustive foraging is similarly organized as exhaustive ones, at least for those factors studied in exhaustive foraging: feature conditions show more organized search patterns compared to conjunction ones (e.g., Kristjánsson et al., 2022), and older observers show more organized patterns compared to younger ones (e.g., Ólafsdóttir et al., 2021; Woods et al., 2013). It is important to note that better organization is related to better search efficiency (Smith & DeLillo, 2022), which is the case in our study. Although not a primary objective in this study, we have verified this fact in Annex 2 for the interested reader: Indeed, in our non-exhaustive motion foraging game, a more organized search results in a more efficient search too.

Importantly, we have also tested two critical variables in the organization not previously studied in the exhaustive foraging literature: the time course of organization (studying changes over time within patches) and potential set size; that is, the fact that not all environments contain the same number of elements to look for (targets) and to avoid (distractors). These manipulations tried to depict a more detailed picture of search organization by answering other critical questions raised in this study: How does organization evolve during the search in diverse (set size) environments? And how does organization differently progress in development?

Although our findings suggest a complex relationship between age, condition, set size, and time (remember that the model including the four-way interaction, was the better model fitting data for all organization indicators tested), we can depict several conclusions from these results. Graphical analyses of the explored models show that, for every foraging indicator and condition, the first time-bin is more organized than the second one: As time goes by within a trial (within a patch), foraging becomes less organized. Wolfe (2013) noted that participants tended to pick up the "easiest" targets first. It seems that the greater target abundance in the beginning of the search helps to better organize the search, according to our results. Remember that targets disappeared as observers picked them up, while distractors remained, increasing the distractor/target ratio as the trial advanced.

Moreover, time-bin effects were clear and constant over the organization indicators (unlike other factors such as set size). That is, search organization decreases when search termination is coming. That organization decrease suggests that we could use organization measures as a potential clue to understanding quitting rules in search. We could use organization as patch leaving criteria, rather than (or complementary to) the rate of targets picked, as used in other models like the Marginal Value Theorem (MVT; Charnov, 1976). Indeed, as shown in the results, these effects seem relatively stable regardless differences in age, set size, or conditions. Gil-Gómez de Liaño et al. (2022) found that search quitting rules based on MVT, follow similar patterns even in children as young as 5 years old, supporting that organization rules could also be a valuable tool to predict quitting behavior in foraging at all age stages. The study of search organization in foraging (and potentially in other types of visual search) seems critical to understand complex cognitive decision processes in search, like those governing quitting rules. The study of organization in search suggests a considerable potentiality of applications in the real world, like in medical image perception. Understanding how a radiologist organizes search during cancer-nodules search in a mammogram could help predict the optimal time to quit the search, maybe with more confidence to reduce potential false positive/negative responses. Future research should devote efforts to testing organization measures to estimate the optimal moment to leave a search in more real-world tasks.

The effects of set size are less clear though. We hypothesized that larger set sizes would result in lower organization due to a shortening of the FVF with larger set sizes (Motter & Simoni, 2008). And this happens for all measures but for Mean ITD, and at some conditions for Best-r and ρ_d_. For the rest of the indexes, a global effect of set size is apparent in the way predicted, with larger set size conditions showing lower levels of organization. For mean ITDs we have seen better organization for shorter set size conditions. Keeping the target proportion approximately constant, as is our case (remember, it could vary between 20% and 30% in every set size condition), a larger set size implies that items (targets or distractors) are closer to each other, showing higher density than shorter set sizes. Those shorter distances among items under larger set sizes could explain why mean ITD decreases without necessarily reflecting more organization (as it measures “intertarget-distances”), explaining the discrepancies for mean ITD results. Also, remember that mean ITD should be cautiously considered in dynamic environments, although the rest of the results replicate those found in exhaustive foraging (e.g., Ólafsdóttir et al., 2021). Furthermore, the effects of set size seem more pronounced in younger participants. This more pronounced effect in younger observers may reflect that younger participants are less capable of inhibiting distractors than older ones, thus being more sensitive to the effect of set size, as also reported in previous studies in visual search (Gil-Gómez de Liaño et al., 2020) and foraging (Gil-Gómez de Liaño & Wolfe, 2022). Furthermore, unlike the other indicators, best-r and ρ_d_ slightly increase with set size for older participants. Best-r and ρ_d_ capture the trend to keep searching in horizontal or vertical lines (Clarke et al., 2022a; Mark et al., 2004). Thus, this strategy might be more intuitive when the display has many targets (easier to follow an up-down and left-right type of search) and that the oldest participants may be more sensitive to this effect, that is, older observers (from about 11–12 years old) are more used to use and have better reading-type skills compared to younger observers (about 4–10 years old). However, more research is needed to support this explanation.

Finally, considering the two spatial indexes described in foraging Bayesian models (Clarke et al., 2022a; the first two parameters are essentially unrelated to the spatial organization, as expected), we found that the preference for picking up closer targets moderately correlates with best-r and strongly with the rest of organization indicators (see Table 3). The correlation between ρ_d_ and mean ITD and PAO is expectable because the ρ_d_ is related to the search of the closest target, and mean ITD and PAO are related to look for the shortest paths. In turn, the small correlation between |ρ_Θ_| and the organization indicators point towards a slight relationship between the persistence in the scan direction and the reading-like patterns detected by best-r or the optimization of path lengths captured by the other indicators. In any case, more research is needed to understand these new Bayesian spatial parameters in foraging. Several models focused on target processing, such as guided visual search (Wolfe, 2021b) or the theory of visual attention (Bundesen, 1990), may provide theoretical frameworks to further study the proximity bias parameters recently described by Clarke et al. (2022a, b) and others (Le et al., 2023; Tünnermann et al., 2022). For the present work, they seem to show interesting results and significant correlations with some organization parameters worth to continue studying and understanding.

In summary, in this study we show evidence that dynamic non-exhaustive foraging is similarly organized than exhaustive, more controlled searches, with easier feature tasks more organized than more difficult, conjunction ones, with age increasing the organization rules (being search more organized for adolescents and adults than for children), and with environments with lower set sizes allowing better search organization. Importantly, at the beginning of the search, the organization tends to be more structured than when time goes by within the search. But later, although less organized, organization indicators could be crucial predictors of quitting rules in search. They could be used as a patch leaving criterion, and future research should address this possibility. Finally, although different, the four organization measures here tested (best-r, mean ITDs, PAO, and intersection rates) share a common variance, which suggests the possibility of developing a joint organization measure in the future, together with the new Bayesian indexes, that seem to correlate with these organization measures. Although more research is needed, we show empirical evidence that the study of organization in complex motion, real world-like foraging tasks can be a potential tool to better understand search processes in humans in the lifespan.

### Supplementary Information

Below is the link to the electronic supplementary material.

Supplementary file1 (DOCX 89 KB)

Supplementary file2 (R 68 KB)

## Data Availability

The database with the organization parameters obtained is publicly available (https://osf.io/k9gj4/).

## Footnotes

1 As in other studies in visual foraging (e.g., Gil-Gómez de Liaño et al., 2022; Ólafsdóttir et al., 2021; Wolfe, 2013; Woods et al., 2013), each trial corresponds to a display/patch in our study too. A display is analogous to a patch of food where an animal forages until food becomes scarce enough to be preferable to travel to the next one (see Bella-Fernández et al., 2022, for a review).

2 We tried to correct the mean ITD for the number of remaining targets in the display, but the results showed no correlations between the corrected mean ITD and the other organization indicators.

3 Np-hard problems are problems without known algorithms to be solved in a reasonable time (or polynomial-time algorithm; see MacGregor & Chu, 2011).

4 Another possible criterion for generating the bins is the duration of the task within a patch, instead of target collection. We also performed those analyses and observed that the correlations between analogous bins for each method were high, ranging from .655 to .915. Thus, both methods yielded to similar results.

## Code availability

The R code used is available as Supplementary Material (https://osf.io/k9gj4/).
