# Supplementary material for: The time course of visual foraging in the lifespan: Spatial scanning, organization search, and target processing
Source: Psychon Bull Rev. 2023 Aug 24;31(1):325–39. doi: 10.3758/s13423-023-02345-8 (PMC10867067; doi:10.3758/s13423-023-02345-8)
Supplement: Supplementary file 1 — Supplementary file1 (DOCX 89 KB) [file 13423_2023_2345_MOESM1_ESM.docx]

**Annex 1: Model Fitting**

As mentioned in the Methods section, Burnham et al. (2011) described a method for comparing competing models. This method is based on the AIC of every model.

Starting from the AIC value for each model, AIC_c_ is calculated as a correction of AIC values for small sample sizes, as follows:

${AIC}_{c}=AIC+(2K\left( K-1 \right))/(n-K-1)$ [S1]

Where *K* is the number of parameters to be estimated for the model, and *n* is the sample size. It is noteworthy that AIC_c_ converges to AIC as *n* becomes larger. The smaller the AIC value, the better the model fit (Burnham et al., 2011).

After that, Δ is calculated for every model *i*:

$\Delta_{i}={AIC}_{c_{i}}-{AIC}_{c_{min}}$ [S2]

AIC_c min_ is the AIC_c_ for the best-fitting model. Δ is a measure of differences between model information. Although there is no strict rules for Δ interpretation, Δ values greater than 20 generally mean that this model loses too much information compared to the full model, so they are expected to be lower, where the lower the better.

Lastly, it is also possible to calculate *l*, which is simply the relative likelihood of each model compared to the best model:

$l=e^{{-\Delta}/2}$ [S3]

These kind of methods for model comparison require careful thinking when proposing plausible models. In our case, analyzing four factors and all their interactions gives a total of 15 effects. Contemplating the presence or absence of each one of these effects would result in 2^15^ possible models. To avoid this, we fixed the presence or absence of a number of effects based on theory. Based on previous results (Ólafsdóttir et al., 2021; Woods et al., 2013) and the polynomial regression models (see Results), we assumed the four simple effects to have coefficients significantly different from 0. We also assumed the two-way Age x Condition interaction to be 0, as well as the three-way interactions. These decisions and the hypotheses described in the introduction led us to anticipate 10 resulting models described in Table 1 in the main manuscript.

**Annex 2: The relationship between organization and efficiency in foraging**

Although there are recent studies demonstrating the relationship between organization and search efficiency, with more organized searches being more efficient (Smith & DeLillo, 2022), this is not necessarily true, especially under non-exhaustive tasks with items in random movement. It could be the case that staying in one particular section of the spatial area waiting for targets to come and pick up could be an effective way of search relying on probably low organization indicators. For that reason, it is important to correlate our organization measures with efficiency measures in the foraging task, to make sure of this relationship between efficiency and organization in our non-exhaustive search. Thus, we estimated the foraging efficiency and accuracy for every display too, in order to correlate these measures with organization measures.

Efficiency can be measured by dividing the total time invested in the display by the proportion of correct hits, what is called Inverse Efficiency Score (IES; Townsend & Ashby, 1983). Furthermore, Geldmacher and Hills (1997) proposed an indicator combining search speed and accuracy in a single measure, the Q score. The Q score is conceived as way to measure the quality of search. High Q score values are related to a good combination of speed and efficacy selecting targets, and therefore a higher efficiency in the search. Q score has been used in cancellation tasks (Dalmaijer et al., 2015), but not directly for visual foraging tasks. Q score is calculated for every trial as follows:

$Q=\frac{n_{col}}{n_{tar}}*\frac{n_{col}}{t_{t}}=\frac{n_{col}^{2}}{n_{tar}*t_{t}}$ [S4]

Where n_col_ is the number of targets collected, n_tar_ is the number of targets on the display (not the set size), and t_t_ is the total search time. To further explore the relationship between foraging organization and efficiency in dynamic foraging, we estimated the correlations between the organization indicators and these foraging efficiency measures.

Table S1 shows the correlation between the organization and the two efficiency indicators described. As we can see, in general, our organization measures are significantly related with efficiency in the way expected, especially for the IES index: Higher levels of efficiency are positively correlated with higher levels of organization (higher values of best-r and lower levels of the rest of the indexes).

Table S1: Pearson correlations between organization indicators and efficiency indicators.

|  | IES^[[1]](#footnote-1)^ | Q score |
| --- | --- | --- |
| Best-r | -.366 (p < .001) | .130 (p > .001) |
| Mean ITD | .288 (p > .001) | -.201 (p > .001) |
| PAO | .599 (p > .001) | -.157 (p > .001) |
| Intersection rate | .624 (p >.001) | -.138 (p > .001) |
| ρ_d_ | -.475 (p >.001) | .285 (p >.001) |
| ρ_Θ_ | .290 (p >.001) | -.197 (p >.001) |

**Annex 3: Descriptive statistics of organization indicators by age, condition, set size, and bin**

Tables S2 to S7 show the means and standard deviations of the four organization variables, as well as the parameters b3 and b4 from the Clarke et al. (2022a) model for each condition from the combination of the four factors age, condition, set size, and bin.

Table S2: Means (sd) of best-r by condition, age, and bin.

| Bin 1 | | | | | | | | | | | | | | | | | | | | | | | |
| --- | --- | --- | --- | --- | --- | --- | --- | --- | --- | --- | --- | --- | --- | --- | --- | --- | --- | --- | --- | --- | --- | --- | --- |
|  | Age / Set Size | 4 | 5 | 6 | 7 | 8 | 9 | 10 | 11 | 12 | 13 | 14 | 15 | 16 | 17 | 18 | 19 | 20 | 21 | 22 | 23 | 24 | 25 |
| Feature | 60 | 0.74 (0.19) | 0.8 (0.13) | 0.78 (0.14) | 0.8 (0.16) | 0.74 (0.23) | 0.81 (0.17) | 0.81 (0.19) | 0.76 (0.14) | 0.76 (0.18) | 0.81 (0.13) | 0.74 (0.2) | 0.8  (0.2) | 0.73 (0.22) | 0.22 (0.18) | 0.79 (0.19) | 0.83 (0.13) | 0.73 (0.29) | 0.76 (0.25) | 0.76 (0.19) | 0.86 (0.06) | 0.91 (NA) | NaN (NA) |
|  | 100 | 0.7  (0.2) | 0.75 (0.18) | 0.71 (0.22) | 0.79 (0.15) | 0.77 (0.16) | 0.69 (0.25) | 0.72 (0.23) | 0.72 (0.22) | 0.65 (0.29) | 0.73 (0.14) | 0.78 (0.22) | 0.76 (0.13) | 0.73 (0.25) | 0.66 (0.46) | 0.65 (0.17) | 0.73 (0.16) | 0.76 (0.18) | 0.68 (0.24) | 0.73 (0.2) | 0.82 (0.12) | 0.67 (NA) | 0.69 (NA) |
|  | 140 | 0.56 (0.25) | 0.62 (0.21) | 0.61 (0.21) | 0.62 (0.23) | 0.67 (0.19) | 0.6 (0.24) | 0.72 (0.19) | 0.75 (0.17) | 0.6 (0.24) | 0.73 (0.2) | 0.73 (0.18) | 0.66 (0.26) | 0.69 (0.24) | 0.74 (0.21) | 0.68 (0.28) | 0.68 (0.14) | 0.8 (0.15) | 0.73 (0.13) | 0.77 (0.06) | NaN (NA) | 0.8 (0.01) | 0.71 (0.08) |
|  | 180 | 0.61 (0.23) | 0.61 (0.22) | 0.65 (0.21) | 0.63 (0.22) | 0.68 (0.2) | 0.69 (0.21) | 0.73 (0.18) | 0.64 (0.23) | 0.66 (0.18) | 0.75 (0.17) | 0.7 (0.17) | 0.7 (0.25) | 0.8 (0.17) | 0.72 (0.32) | 0.74 (0.18) | 0.73 (0.19) | 0.64 (0.3) | 0.83 (0.11) | 0.87 (0.09) | 0.63 (0.08) | 0.82 (0.13) | 0.73 (0.23) |
| Conjunction | 60 | 0.78 (0.2) | 0.75 (0.21) | 0.77 (0.19) | 0.72 (0.22) | 0.68 (0.24) | 0.72 (0.21) | 0.75 (0.2) | 0.8 (0.16) | 0.76 (0.17) | 0.75 (0.21) | 0.79 (0.16) | 0.84 (0.15) | 0.8 (0.21) | 0.85 (0.07) | 0.93 (0.05) | 0.8 (0.13) | 0.94 (0.06) | 0.8 (0.11) | 0.88 (0.16) | 0.76 (NA) | 0.84 (0.05) | NaN (NA) |
|  | 100 | 0.65 (0.23) | 0.62 (0.24) | 0.6 (0.27) | 0.63 (0.22) | 0.71 (0.2) | 0.65 (0.23) | 0.69 (0.21) | 0.71 (0.17) | 0.62 (0.28) | 0.7 (0.22) | 0.74 (0.24) | 0.66 (0.23) | 0.7 (0.17) | 0.71 (0.29) | 0.79 (0.16) | 0.75 (0.18) | 0.71 (0.13) | 0.66 (0.25) | 0.51 (0.25) | 0.21 (NA) | 0.7 (0.13) | 0.73 (0.23) |
|  | 140 | 0.58 (0.22) | 0.51 (0.22) | 0.58 (0.24) | 0.63 (0.27) | 0.61 (0.18) | 0.58 (0.23) | 0.61 (0.24) | 0.82 (0.14) | 0.41 (0.15) | 0.98 (0) | 0.72 (0.24) | 0.94 (0.04) | 0.55 (0.09) | NaN (NA) | 0.67 (0.17) | 0.6 (0.24) | 0.7  (0.3) | 0.45 (0.2) | 0.53 (0.18) | 0.6 (0.21) | NaN (NA) | NaN (NA) |
|  | 180 | 0.6 (0.24) | 0.54 (0.24) | 0.6 (0.22) | 0.59 (0.25) | 0.6 (0.19) | 0.57 (0.19) | 0.65 (0.22) | 0.67 (0.19) | 0.59 (0.25) | 0.72 (0.22) | 0.74 (0.21) | 0.64 (0.24) | 0.73 (0.17) | 0.58 (0.35) | 0.62 (0.26) | 0.6  (0.2) | 0.58 (0.24) | 0.69 (0.23) | 0.66 (0.19) | 0.55 (0.23) | 0.41 (NA) | 0.8 (0.23) |
| Bin 2 | | | | | | | | | | | | | | | | | | | | | | | |
|  | Age / Set Size | 4 | 5 | 6 | 7 | 8 | 9 | 10 | 11 | 12 | 13 | 14 | 15 | 16 | 17 | 18 | 19 | 20 | 21 | 22 | 23 | 24 | 25 |
| Feature | 60 | 0.62 (0.24) | 0.52 (0.25) | 0.59 (0.25) | 0.53 (0.23) | 0.57 (0.32) | 0.62 (0.26) | 0.66 (0.26) | 0.51 (0.29) | 0.5 (0.32) | 0.62 (0.27) | 0.66 (0.29) | 0.66 (0.28) | 0.45 (0.28) | 0.46 (0.46) | 0.43 (0.34) | 0.56 (0.26) | 0.64 (0.12) | 0.6 (0.31) | 0.71 (0.21) | 0.91 (0.1) | 0.84 (NA) | NaN (NA) |
|  | 100 | 0.51 (0.29) | 0.52 (0.22) | 0.54 (0.28) | 0.61 (0.23) | 0.65 (0.23) | 0.59 (0.28) | 0.64 (0.24) | 0.65 (0.27) | 0.66 (0.15) | 0.72 (0.19) | 0.77 (0.2) | 0.68 (0.23) | 0.68 (0.25) | 0.77 (0.12) | 0.65 (0.23) | 0.67 (0.25) | 0.47 (0.28) | 0.6 (0.31) | 0.68 (0.18) | 0.68 (0.28) | 0.82 (NA) | 0.72 (NA) |
|  | 140 | 0.43 (0.23) | 0.43 (0.22) | 0.46 (0.27) | 0.51 (0.26) | 0.42 (0.22) | 0.5 (0.25) | 0.6 (0.19) | 0.56 (0.22) | 0.5 (0.25) | 0.6 (0.21) | 0.64 (0.22) | 0.55 (0.25) | 0.5 (0.31) | 0.55 (0.2) | 0.65 (0.26) | 0.59 (0.15) | 0.49 (0.25) | 0.64 (0.25) | 0.61 (0.35) | NaN (NA) | 0.63 (0.06) | 0.55 (0.32) |
|  | 180 | 0.48 (0.23) | 0.54 (0.22) | 0.48 (0.22) | 0.46 (0.25) | 0.55 (0.23) | 0.52 (0.22) | 0.56 (0.22) | 0.53 (0.28) | 0.51 (0.17) | 0.62 (0.25) | 0.64 (0.27) | 0.62 (0.17) | 0.49 (0.15) | 0.69 (0.1) | 0.67 (0.28) | 0.5 (0.19) | 0.57 (0.22) | 0.71 (0.24) | 0.64 (0.24) | 0.49 (0.21) | 0.69 (0.06) | 0.41 (0.4) |
| Conjunction | 60 | 0.66 (0.23) | 0.66 (0.22) | 0.62 (0.28) | 0.7 (0.21) | 0.59 (0.25) | 0.62 (0.25) | 0.63 (0.23) | 0.65 (0.24) | 0.6 (0.24) | 0.75 (0.22) | 0.63 (0.25) | 0.68 (0.24) | 0.65 (0.24) | 0.67 (0.24) | 0.7 (0.04) | 0.53 (0.34) | 0.57 (0.27) | 0.59 (0.18) | 0.48 (0.4) | 0.15 (NA) | 0.87 (0.06) | NaN (NA) |
|  | 100 | 0.52 (0.26) | 0.57 (0.22) | 0.48 (0.24) | 0.52 (0.26) | 0.54 (0.27) | 0.52 (0.25) | 0.56 (0.27) | 0.62 (0.2) | 0.59 (0.25) | 0.57 (0.25) | 0.57 (0.25) | 0.56 (0.26) | 0.62 (0.26) | 0.62 (0.22) | 0.5 (0.28) | 0.63 (0.25) | 0.55 (0.26) | 0.71 (0.21) | 0.66 (0.28) | 0.71 (NA) | 0.74 (0.32) | 0.26 (0.34) |
|  | 140 | 0.55 (0.26) | 0.41 (0.27) | 0.58 (0.25) | 0.51 (0.25) | 0.38 (0.19) | 0.46 (0.25) | 0.48 (0.27) | 0.74 (0.08) | 0.36 (0.19) | 0.55 (0.32) | 0.47 (0.26) | 0.72 (0.28) | 0.47 (0.11) | NaN (NA) | 0.48 (0.22) | 0.56 (0.27) | 0.5 (0.18) | 0.45 (0.16) | 0.57 (0.17) | 0.54 (0.37) | NaN (NA) | NaN (NA) |
|  | 180 | 0.48 (0.24) | 0.57 (0.25) | 0.43 (0.23) | 0.46 (0.22) | 0.59 (0.2) | 0.49 (0.24) | 0.44 (0.24) | 0.53 (0.25) | 0.49 (0.27) | 0.59 (0.24) | 0.6 (0.27) | 0.55 (0.25) | 0.62 (0.23) | 0.34 (0.27) | 0.57 (0.35) | 0.56 (0.24) | 0.47 (0.28) | 0.63 (0.29) | 0.6 (0.19) | 0.4 (0.16) | 0.11 (NA) | 0.65 (0.25) |

Table S3: Means (sd) of Mean ITD by condition, age, and bin.

| Bin 1 | | | | | | | | | | | | | | | | | | | | | | | |
| --- | --- | --- | --- | --- | --- | --- | --- | --- | --- | --- | --- | --- | --- | --- | --- | --- | --- | --- | --- | --- | --- | --- | --- |
|  | Age / Set Size | 4 | 5 | 6 | 7 | 8 | 9 | 10 | 11 | 12 | 13 | 14 | 15 | 16 | 17 | 18 | 19 | 20 | 21 | 22 | 23 | 24 | 25 |
| Feature | 60 | 260 (89) | 252 (81) | 267 (118) | 293 (110) | 257 (73) | 270 (87) | 274 (90) | 320 (66) | 301 (111) | 301 (81) | 291 (93) | 304 (62) | 328 (59) | 360 (101) | 271 (179) | 320 (121) | 265 (102) | 323 (182) | 366 (60) | 282 (106) | 230 (NA) | NaN (NA) |
|  | 100 | 248 (70) | 220 (69) | 255 (97) | 245 (72) | 219 (54) | 216 (67) | 240 (87) | 236 (50) | 227 (59) | 247 (63) | 208 (57) | 253 (80) | 216 (56) | 309 (31) | 239 (72) | 255 (96) | 279 (112) | 222 (73) | 205 (51) | 205 (64) | 182 (NA) | 268 (NA) |
|  | 140 | 276 (65) | 273 (69) | 272 (77) | 264 (67) | 246 (53) | 246 (59) | 222 (54) | 215 (49) | 241 (75) | 205 (48) | 216 (65) | 224 (48) | 216 (49) | 231 (47) | 229 (47) | 228 (68) | 187 (39) | 206 (48) | 205 (38) | NaN (NA) | 184 (34) | 194 (14) |
|  | 180 | 239 (54) | 244 (69) | 244 (53) | 238 (44) | 227 (59) | 232 (57) | 207 (60) | 237 (40) | 258 (61) | 224 (57) | 225 (59) | 222 (51) | 213 (44) | 268 (127) | 219 (91) | 221 (36) | 214 (30) | 198 (42) | 196 (65) | 233 (105) | 179 (11) | 201 (87) |
| Conjunction | 60 | 294 (90) | 289 (102) | 248 (105) | 311 (114) | 307 (104) | 296 (107) | 273 (84) | 271 (90) | 310 (57) | 286 (98) | 294 (75) | 281 (61) | 277 (105) | 291 (62) | 298 (66) | 297 (88) | 292 (45) | 298 (123) | 368 (217) | 321 (NA) | 306 (102) | NaN (NA) |
|  | 100 | 293 (88) | 304 (95) | 294 (81) | 320 (114) | 292 (107) | 289 (97) | 279 (90) | 265 (77) | 259 (86) | 271 (90) | 267 (95) | 286 (86) | 292 (94) | 296 (84) | 228 (64) | 272 (80) | 295 (53) | 301 (99) | 313 (111) | 164 (NA) | 314 (106) | 281 (3) |
|  | 140 | 298 (84) | 303 (91) | 289 (89) | 324 (88) | 314 (70) | 312 (62) | 277 (62) | 284 (191) | 331 (67) | 236 (58) | 248 (110) | 265 (45) | 251 (47) | NaN (NA) | 256 (86) | 271 (102) | 286 (113) | 324 (61) | 295 (134) | 236 (114) | NaN (NA) | NaN (NA) |
|  | 180 | 281 (99) | 307 (70) | 262 (61) | 294 (96) | 264 (59) | 272 (86) | 267 (83) | 273 (110) | 283 (107) | 245 (91) | 234 (65) | 255 (54) | 223 (48) | 274 (103) | 266 (79) | 238 (47) | 256 (81) | 254 (60) | 237 (45) | 270 (84) | 243 (NA) | 212 (45) |
| Bin 2 | | | | | | | | | | | | | | | | | | | | | | | |
|  | Age / Set Size | 4 | 5 | 6 | 7 | 8 | 9 | 10 | 11 | 12 | 13 | 14 | 15 | 16 | 17 | 18 | 19 | 20 | 21 | 22 | 23 | 24 | 25 |
| Feature | 60 | 311 (104) | 405 (106) | 381 (105) | 423 (122) | 373 (139) | 391 (99) | 363 (101) | 330 (98) | 362 (111) | 361 (90) | 354 (119) | 386 (144) | 393 (131) | 482 (62) | 448 (109) | 369 (85) | 410 (81) | 366 (67) | 263 (85) | 276 (51) | 442 (NA) | NaN (NA) |
|  | 100 | 349 (91) | 415 (105) | 393 (105) | 396 (105) | 358 (100) | 373 (70) | 369 (109) | 381 (92) | 410 (113) | 386 (132) | 297 (71) | 365 (103) | 359 (146) | 298 (58) | 361 (95) | 356 (113) | 394 (174) | 377 (126) | 337 (76) | 375 (124) | 348 (NA) | 274 (NA) |
|  | 140 | 373 (98) | 385 (90) | 367 (91) | 390 (92) | 419 (96) | 401 (103) | 352 (105) | 377 (113) | 432 (117) | 379 (96) | 340 (89) | 378 (98) | 410 (142) | 375 (143) | 341 (101) | 402 (93) | 389 (110) | 372 (105) | 311 (61) | NaN (NA) | 313 (11) | 274 (17) |
|  | 180 | 318 (68) | 332 (79) | 348 (85) | 391 (71) | 332 (75) | 354 (74) | 332 (62) | 361 (62) | 372 (80) | 352 (90) | 302 (78) | 347 (99) | 377 (82) | 288 (142) | 268 (79) | 387 (57) | 330 (63) | 343 (126) | 356 (90) | 336 (30) | 382 (1) | 441 (180) |
| Conjunction | 60 | 327 (99) | 395 (103) | 331 (96) | 385 (100) | 374 (98) | 402 (107) | 362 (105) | 304 (102) | 365 (79) | 328 (113) | 331 (98) | 323 (113) | 319 (84) | 365 (37) | 328 (65) | 300 (67) | 399 (96) | 422 (90) | 362 (39) | 556 (NA) | 422 (47) | NaN (NA) |
|  | 100 | 362 (115) | 373 (104) | 393 (112) | 432 (89) | 391 (102) | 405 (128) | 381 (99) | 376 (79) | 357 (106) | 358 (101) | 372 (95) | 389 (90) | 354 (101) | 414 (121) | 408 (126) | 364 (145) | 369 (92) | 357 (103) | 334 (81) | 339 (NA) | 344 (104) | 397 (52) |
|  | 140 | 345 (97) | 363 (94) | 358 (106) | 405 (95) | 391 (104) | 408 (55) | 375 (101) | 378 (126) | 359 (128) | 229 (1) | 357 (98) | 287 (157) | 366 (144) | NaN (NA) | 416 (92) | 387 (97) | 396 (72) | 360 (130) | 341 (112) | 370 (127) | NaN (NA) | NaN (NA) |
|  | 180 | 345 (111) | 386 (97) | 365 (87) | 414 (87) | 333 (85) | 386 (93) | 365 (81) | 352 (89) | 340 (72) | 334 (52) | 338 (83) | 347 (69) | 332 (76) | 423 (89) | 370 (39) | 321 (72) | 340 (40) | 315 (79) | 328 (32) | 395 (21) | 389 (NA) | 381 (32) |

Table S4: Means (sd) of PAO by condition, age, and bin.

| Bin 1 | | | | | | | | | | | | | | | | | | | | | | | |
| --- | --- | --- | --- | --- | --- | --- | --- | --- | --- | --- | --- | --- | --- | --- | --- | --- | --- | --- | --- | --- | --- | --- | --- |
|  | Age / Set Size | 4 | 5 | 6 | 7 | 8 | 9 | 10 | 11 | 12 | 13 | 14 | 15 | 16 | 17 | 18 | 19 | 20 | 21 | 22 | 23 | 24 | 25 |
| Feature | 60 | 34 (34) | 26 (32) | 24 (18) | 22 (28) | 26 (29) | 19 (23) | 16 (13) | 21 (13) | 24 (20) | 19 (14) | 22 (20) | 14 (9) | 25 (17) | 27 (9) | 24 (0) | 22 (12) | 25 (31) | 22 (22) | 26 (2) | 21 (16) | 0 (NA) | NaN (NA) |
|  | 100 | 31 (21) | 26 (23) | 30 (26) | 28 (29) | 23 (14) | 32 (26) | 27 (24) | 20 (15) | 31 (20) | 22 (11) | 26 (20) | 22 (16) | 36 (25) | 48 (42) | 30 (19) | 37 (34) | 44 (44) | 23 (23) | 17 (13) | 31 (20) | 43 (NA) | 54 (NA) |
|  | 140 | 48 (29) | 42 (26) | 33 (30) | 37 (24) | 33 (28) | 35 (29) | 25 (28) | 24 (23) | 44 (32) | 28 (26) | 26 (26) | 33 (30) | 27 (24) | 24 (19) | 35 (36) | 40 (36) | 18 (18) | 29 (23) | 25 (17) | NaN (NA) | 7 (5) | 13 (4) |
|  | 180 | 40 (29) | 44 (24) | 37 (23) | 28 (24) | 32 (26) | 34 (21) | 24 (18) | 36 (21) | 54 (45) | 33 (26) | 26 (27) | 43 (35) | 32 (18) | 23 (39) | 31 (26) | 40 (30) | 43 (21) | 21 (16) | 19 (14) | 59 (31) | 11 (12) | 34 (30) |
| Conjunction | 60 | 24 (21) | 26 (21) | 30 (32) | 24 (23) | 28 (27) | 25 (22) | 26 (22) | 21 (17) | 27 (24) | 21 (21) | 22 (16) | 23 (20) | 14 (16) | 27 (24) | 4 (6) | 32 (24) | 0 (0) | 14 (13) | 31 (35) | 34 (NA) | -1 (7) | NaN (NA) |
|  | 100 | 44 (37) | 42 (27) | 44 (31) | 42 (30) | 35 (31) | 35 (24) | 32 (31) | 26 (18) | 39 (38) | 27 (28) | 29 (25) | 34 (27) | 33 (28) | 38 (52) | 15 (13) | 31 (27) | 26 (16) | 38 (27) | 43 (14) | 33 (NA) | 17 (20) | 19 (27) |
|  | 140 | 54 (28) | 45 (29) | 49 (33) | 31 (23) | 39 (36) | 47 (34) | 34 (22) | 25 (22) | 60 (29) | 0 (0) | 43 (53) | 15 (18) | 34 (23) | NaN (NA) | 53 (36) | 50 (34) | 48 (61) | 48 (35) | 53 (49) | 38 (46) | NaN (NA) | NaN (NA) |
|  | 180 | 47 (35) | 55 (29) | 40 (29) | 44 (31) | 41 (23) | 49 (28) | 38 (29) | 35 (21) | 38 (24) | 33 (24) | 21 (15) | 43 (28) | 22 (13) | 38 (44) | 35 (27) | 41 (31) | 40 (25) | 34 (19) | 34 (26) | 58 (40) | 71 (NA) | 16 (28) |
| Bin 2 | | | | | | | | | | | | | | | | | | | | | | | |
|  | Age / Set Size | 4 | 5 | 6 | 7 | 8 | 9 | 10 | 11 | 12 | 13 | 14 | 15 | 16 | 17 | 18 | 19 | 20 | 21 | 22 | 23 | 24 | 25 |
| Feature | 60 | 48 (40) | 47 (29) | 48 (23) | 42 (28) | 45 (39) | 45 (34) | 36 (40) | 63 (41) | 59 (40) | 44 (50) | 54 (42) | 56 (53) | 74 (58) | 114 (27) | 100 (76) | 52 (19) | 94 (87) | 55 (38) | 39 (6) | 42 (6) | 43 (NA) | NaN (NA) |
|  | 100 | 52 (36) | 67 (44) | 74 (64) | 52 (45) | 46 (31) | 51 (45) | 57 (51) | 41 (34) | 62 (47) | 44 (34) | 25 (22) | 36 (42) | 64 (97) | 26 (12) | 46 (37) | 53 (51) | 112 (136) | 83 (120) | 46 (30) | 62 (69) | 55 (NA) | 27 (NA) |
|  | 140 | 103 (69) | 90 (58) | 69 (43) | 74 (51) | 87 (59) | 84 (75) | 63 (70) | 64 (61) | 137 (142) | 56 (52) | 69 (101) | 104 (142) | 99 (104) | 64 (68) | 54 (73) | 151 (111) | 95 (123) | 85 (108) | 29 (50) | NaN (NA) | 47 (17) | 26 (24) |
|  | 180 | 72 (44) | 73 (44) | 78 (37) | 91 (54) | 63 (38) | 75 (42) | 60 (38) | 93 (80) | 114 (110) | 65 (51) | 79 (120) | 69 (56) | 73 (40) | 61 (41) | 49 (35) | 100 (57) | 74 (31) | 74 (77) | 63 (26) | 92 (9) | 71 (24) | 111 (89) |
| Conjunction | 60 | 40 (31) | 44 (33) | 37 (29) | 39 (30) | 42 (32) | 39 (28) | 40 (32) | 54 (27) | 44 (24) | 30 (22) | 38 (24) | 52 (42) | 31 (25) | 53 (36) | 19 (15) | 65 (30) | 87 (51) | 60 (33) | 62 (55) | 138 (NA) | 8 (4) | NaN (NA) |
|  | 100 | 65 (38) | 66 (42) | 64 (37) | 68 (49) | 56 (43) | 58 (43) | 51 (43) | 53 (26) | 63 (50) | 56 (36) | 77 (74) | 75 (64) | 56 (55) | 56 (48) | 76 (70) | 59 (85) | 62 (41) | 47 (43) | 30 (30) | 39 (NA) | 23 (26) | 70 (53) |
|  | 140 | 58 (42) | 69 (48) | 73 (61) | 55 (38) | 81 (44) | 76 (38) | 73 (62) | 49 (25) | 95 (76) | 44 (15) | 80 (75) | 35 (30) | 82 (66) | NaN (NA) | 133 (59) | 86 (58) | 101 (135) | 133 (95) | 67 (51) | 62 (51) | NaN (NA) | NaN (NA) |
|  | 180 | 64 (42) | 69 (44) | 78 (46) | 74 (33) | 69 (42) | 80 (53) | 79 (55) | 53 (32) | 70 (40) | 54 (40) | 50 (38) | 77 (40) | 62 (50) | 70 (64) | 64 (50) | 69 (32) | 81 (48) | 52 (41) | 64 (33) | 105 (38) | 60 (NA) | 77 (67) |

Table S5: Means (sd) of Intersection Rate by condition, age, and bin.

| Bin 1 | | | | | | | | | | | | | | | | | | | | | | | | | | | | | | |
| --- | --- | --- | --- | --- | --- | --- | --- | --- | --- | --- | --- | --- | --- | --- | --- | --- | --- | --- | --- | --- | --- | --- | --- | --- | --- | --- | --- | --- | --- | --- |
|  | Age / Set Size | 4 | | 5 | | 6 | | 7 | | 8 | | 9 | | 10 | | 11 | 12 | 13 | 14 | 15 | 16 | 17 | 18 | 19 | 20 | 21 | 22 | 23 | 24 | 25 |
| Feature | 60 | 0.12 (0.16) | | 0.08 (0.15) | | 0.02 (0.06) | | 0.03 (0.06) | | 0.08 (0.14) | | 0.04 (0.07) | | 0.03 (0.08) | | 0.02 (0.08) | 0.11 (0.12) | 0.02 (0.06) | 0.09 (0.15) | 0.04 (0.08) | 0.08 (0.11) | 0.21 (0.06) | 0 (0) | 0.06 (0.12) | 0.16 (0.28) | 0.04 (0.1) | 0 (0) | 0 (0) | 0 (NA) | NaN (NA) |
|  | 100 | 0.09 (0.11) | | 0.07 (0.11) | | 0.11 (0.14) | | 0.1 (0.11) | | 0.06 (0.1) | | 0.09 (0.11) | | 0.09 (0.16) | | 0.02 (0.05) | 0.08 (0.19) | 0.03 (0.07) | 0.04 (0.1) | 0.08 (0.1) | 0.06 (0.15) | 0.14 (0.25) | 0.06 (0.1) | 0.06 (0.11) | 0.13 (0.27) | 0.07 (0.12) | 0.04 (0.07) | 0.08 (0.1) | 0.12 (NA) | 0 (NA) |
|  | 140 | 0.2 (0.17) | | 0.17 (0.18) | | 0.15 (0.18) | | 0.16 (0.14) | | 0.14 (0.16) | | 0.17 (0.23) | | 0.11 (0.23) | | 0.08 (0.12) | 0.17 (0.17) | 0.13 (0.17) | 0.1 (0.14) | 0.13 (0.15) | 0.1 (0.13) | 0.11 (0.1) | 0.14 (0.22) | 0.11 (0.16) | 0.04 (0.07) | 0.13 (0.14) | 0.07 (0.09) | NaN (NA) | 0.08 (0.12) | 0.06 (0.09) |
|  | 180 | 0.17 (0.16) | | 0.24 (0.2) | | 0.2 (0.17) | | 0.14 (0.12) | | 0.17 (0.16) | | 0.15 (0.15) | | 0.13 (0.14) | | 0.08 (0.12) | 0.21 (0.25) | 0.12 (0.13) | 0.07 (0.11) | 0.15 (0.13) | 0.13 (0.18) | 0.07 (0.13) | 0.1 (0.1) | 0.21 (0.2) | 0.27 (0.17) | 0.1 (0.09) | 0.02 (0.05) | 0.23 (0.06) | 0.14 (0.04) | 0.11 (0.01) |
| Conjunction | 60 | 0.08 (0.13) | | 0.06 (0.11) | | 0.04 (0.11) | | 0.07 (0.14) | | 0.1 (0.17) | | 0.08 (0.11) | | 0.07 (0.13) | | 0.05 (0.1) | 0.04 (0.09) | 0.04 (0.08) | 0.06 (0.1) | 0.05 (0.1) | 0.07 (0.12) | 0 (0) | 0 (0) | 0.1 (0.15) | 0 (0) | 0 (0) | 0 (0) | 0.2 (NA) | 0 (0) | NaN (NA) |
|  | 100 | 0.17 (0.19) | | 0.18 (0.2) | | 0.17 (0.19) | | 0.15 (0.21) | | 0.12 (0.2) | | 0.12 (0.16) | | 0.12 (0.19) | | 0.11 (0.14) | 0.14 (0.21) | 0.06 (0.12) | 0.11 (0.16) | 0.14 (0.16) | 0.11 (0.21) | 0.07 (0.15) | 0.05 (0.07) | 0.07 (0.11) | 0.05 (0.08) | 0.07 (0.11) | 0.1 (0.18) | 0.17 (NA) | 0.07 (0.1) | 0 (0) |
|  | 140 | 0.21 (0.22) | | 0.22 (0.23) | | 0.22 (0.23) | | 0.13 (0.14) | | 0.16 (0.2) | | 0.18 (0.23) | | 0.08 (0.11) | | 0.03 (0.07) | 0.38 (0.26) | 0 (0) | 0.18 (0.33) | 0 (0) | 0.16 (0.12) | NaN (NA) | 0.19 (0.19) | 0.23 (0.23) | 0.2 (0.31) | 0.19 (0.21) | 0.2 (0.27) | 0.17 (0.35) | NaN (NA) | NaN (NA) |
|  | 180 | 0.22 (0.29) | | 0.23 (0.19) | | 0.17 (0.16) | | 0.2 (0.17) | | 0.19 (0.19) | | 0.24 (0.19) | | 0.16 (0.18) | | 0.13 (0.13) | 0.14 (0.13) | 0.14 (0.12) | 0.12 (0.14) | 0.15 (0.17) | 0.08 (0.08) | 0.19 (0.33) | 0.05 (0.06) | 0.09 (0.09) | 0.15 (0.1) | 0.11 (0.13) | 0.13 (0.13) | 0.23 (0.21) | 0.11 (NA) | 0.16 (0.27) |
| Bin 2 | | | | | | | | | | | | | | | | | | | | | | | | | | | | | | |
|  | Age / Set Size | 4 | 5 | | 6 | | 7 | | 8 | | 9 | | 10 | | 11 | 12 | | 13 | 14 | 15 | 16 | 17 | 18 | 19 | 20 | 21 | 22 | 23 | 24 | 25 |
| Feature | 60 | 0.13 (0.26) | 0.17 (0.18) | | 0.12 (0.16) | | 0.18 (0.17) | | 0.13 (0.2) | | 0.08 (0.12) | | 0.09 (0.14) | | 0.09 (0.13) | 0.18  (0.18) | | 0.08 (0.12) | 0.11 (0.12) | 0.06 (0.13) | 0.21 (0.19) | 0.2 (0.08) | 0.2 (0.28) | 0 (0) | 0.23 (0.36) | 0.02 (0.06) | 0.1 (0.14) | 0 (0) | 0 (NA) | NaN (NA) |
|  | 100 | 0.26 (0.23) | 0.21 (0.23) | | 0.3 (0.34) | | 0.17 (0.19) | | 0.15 (0.15) | | 0.22 (0.22) | | 0.17 (0.2) | | 0.15 (0.2) | 0.14  (0.15) | | 0.15 (0.2) | 0.11 (0.15) | 0.12 (0.16) | 0.23 (0.29) | 0.05 (0.08) | 0.21 (0.26) | 0.24 (0.27) | 0.39 (0.52) | 0.27 (0.38) | 0.12 (0.13) | 0.12 (0.17) | 0.44 (NA) | 0.17 (NA) |
|  | 140 | 0.48 (0.4) | 0.44 (0.33) | | 0.32 (0.22) | | 0.29 (0.26) | | 0.43 (0.39) | | 0.4 (0.36) | | 0.26 (0.36) | | 0.21 (0.22) | 0.58  (0.68) | | 0.23 (0.25) | 0.23 (0.42) | 0.34 (0.52) | 0.37 (0.42) | 0.18 (0.21) | 0.13 (0.26) | 0.37 (0.43) | 0.29 (0.55) | 0.29 (0.42) | 0.12 (0.18) | NaN (NA) | 0.22 (0.31) | 0.06 (0.09) |
|  | 180 | 0.38 (0.26) | 0.39 (0.29) | | 0.45 (0.31) | | 0.43 (0.28) | | 0.31 (0.23) | | 0.4 (0.31) | | 0.33 (0.27) | | 0.4 (0.4) | 0.47  (0.59) | | 0.31 (0.3) | 0.28 (0.4) | 0.38 (0.36) | 0.27 (0.21) | 0.12 (0.11) | 0.16 (0.18) | 0.53 (0.31) | 0.26 (0.18) | 0.42 (0.44) | 0.19 (0.19) | 0.7 (0.24) | 0.25 (0.08) | 0.31 (0.19) |
| Conjunction | 60 | 0.11 (0.16) | 0.2 (0.32) | | 0.13 (0.15) | | 0.11 (0.14) | | 0.17 (0.19) | | 0.12 (0.14) | | 0.11 (0.18) | | 0.11 (0.13) | 0.08  (0.1) | | 0.06 (0.11) | 0.09 (0.2) | 0.08 (0.17) | 0.12 (0.15) | 0.07 (0.12) | 0 (0) | 0.18 (0.18) | 0.12 (0.18) | 0.13 (0.18) | 0.13 (0.23) | 0.33 (NA) | 0 (0) | NaN (NA) |
|  | 100 | 0.28 (0.28) | 0.25 (0.31) | | 0.26 (0.27) | | 0.28 (0.3) | | 0.24 (0.29) | | 0.3 (0.35) | | 0.23 (0.3) | | 0.2 (0.15) | 0.26  (0.31) | | 0.22 (0.21) | 0.3 (0.36) | 0.31 (0.36) | 0.17 (0.24) | 0.19 (0.23) | 0.33 (0.39) | 0.27 (0.51) | 0.14 (0.18) | 0.15 (0.21) | 0.17 (0.23) | 0 (NA) | 0.11 (0.16) | 0.26 (0.2) |
|  | 140 | 0.28 (0.24) | 0.3 (0.27) | | 0.31 (0.32) | | 0.27 (0.24) | | 0.49 (0.45) | | 0.39 (0.3) | | 0.29 (0.33) | | 0.12 (0.11) | 0.58  (0.92) | | 0.07 (0.1) | 0.35 (0.43) | 0.06 (0.1) | 0.56 (0.8) | NaN (NA) | 0.7 (0.36) | 0.39 (0.38) | 0.36 (0.29) | 0.53 (0.39) | 0.29 (0.29) | 0.34 (0.33) | NaN (NA) | NaN (NA) |
|  | 180 | 0.26 (0.22) | 0.33 (0.32) | | 0.38 (0.33) | | 0.38 (0.26) | | 0.36 (0.26) | | 0.4 (0.32) | | 0.48 (0.33) | | 0.24 (0.23) | 0.34  (0.32) | | 0.22 (0.27) | 0.28 (0.32) | 0.37 (0.32) | 0.38 (0.36) | 0.5 (0.84) | 0.37 (0.47) | 0.46 (0.31) | 0.53 (0.42) | 0.23 (0.23) | 0.22 (0.23) | 0.91 (0.25) | 0.2 (NA) | 0.35 (0.47) |

Table S6: Means (sd) of ρ_d_ by condition, age, and bin.

| Bin 1 | | | | | | | | | | | | | | | | | | | | | | | |
| --- | --- | --- | --- | --- | --- | --- | --- | --- | --- | --- | --- | --- | --- | --- | --- | --- | --- | --- | --- | --- | --- | --- | --- |
|  | Age / Set Size | 4 | 5 | 6 | 7 | 8 | 9 | 10 | 11 | 12 | 13 | 14 | 15 | 16 | 17 | 18 | 19 | 20 | 21 | 22 | 23 | 24 | 25 |
| Feature | 60 | 12.2 (6.7) | 17.8 (7.1) | 18.5 (6.4) | 14.4 (9) | 18 (6.4) | 19.3 (4.5) | 19.1 (3.2) | 16 (5.9) | 17.7 (5.4) | 15.4 (6.9) | 16.3 (7) | 19.2 (3.9) | 18.2 (5.5) | 10.8 (14.5) | 18.3 (4.8) | 20.7 (NA) | 20.4 (2) | 18.1 (4.2) | 22.2 (0.4) | NaN (NA) | 22.7 (NA) | 21.2 (NA) |
|  | 100 | 15 (6) | 16.4 (7.7) | 15.3 (3.2) | 19.9 (7.3) | 19.8 (5.2) | 18.2 (7.7) | 21.6 (6) | 21.3 (4.6) | 25 (NA) | 21.5 (NA) | 20.2 (5.2) | 18.7 (8.7) | 17 (8) | NaN (NA) | 19.9 (6.7) | 18.9 (9.4) | 23.5 (3.8) | 23.6 (2.7) | 20.8 (4) | NaN (NA) | 26.2 (NA) | NaN (NA) |
|  | 140 | 10.8 (5.9) | 13.2 (5) | 14.1 (6.7) | 14.8 (6.3) | 16.3 (6.9) | 14.2 (6.5) | 18.3 (7) | 18.6 (6.3) | 16.7 (6.5) | 19.2 (5.8) | 19.7 (5.5) | 18.1 (7.4) | 19.4 (6.6) | 18 (6.2) | 18.7 (6.7) | 16.7 (8.4) | 21.1 (4.3) | 19.8 (5.7) | 21.5 (5.9) | NaN (NA) | 24.8 (4.4) | 22.7 (3.8) |
|  | 180 | 12.1 (6.2) | 13.5 (4.2) | 14 (8) | 16.2 (6.3) | 16.7 (6.1) | 17.1 (5.8) | 20 (7.2) | 14.8 (3.8) | 15.1 (7.9) | 17.9 (7.4) | 19.1 (6.6) | 17 (7.3) | 19.4 (4.6) | 17.4 (11) | 16.7 (6.4) | 14.2 (5.4) | 16.4 (5.7) | 21 (3.9) | 24.7 (3) | NaN (NA) | 27 (0.5) | 21.8 (11.7) |
| Conjunction | 60 | 17 (5.1) | 18.8 (5.2) | 19.2 (7.6) | 15.5 (9.1) | 4.4 (4.5) | 20.4 (1.9) | 14 (8) | 16.7 (8.8) | NaN (NA) | NaN (NA) | 17.3 (NA) | NaN (NA) | NaN (NA) | NaN (NA) | NaN (NA) | 10.1 (11.2) | 22.4 (NA) | NaN (NA) | 15.1 (NA) | 6.4 (NA) | NaN (NA) | NaN (NA) |
|  | 100 | 12.9 (7) | 13.1 (6.9) | 13.6 (8.1) | 14.6 (7.6) | 16.8 (6) | 15 (8) | 9 (9.4) | NaN (NA) | 13.4 (5.7) | 11.3 (4.9) | NaN (NA) | NaN (NA) | NaN (NA) | NaN (NA) | 22.4 (5.6) | 17.1 (5.9) | 14.8 (6.2) | 12.2 (6.9) | 22 (2.7) | 13.9 (NA) | NaN (NA) | NaN (NA) |
|  | 140 | 7.7 (5.9) | 9.7 (6.5) | 11.3 (7.4) | 16.1 (7.1) | 12.9 (7.8) | 14.9 (8.3) | 13 (6.6) | 8 (12.8) | 8 (7.7) | NaN (NA) | NaN (NA) | NaN (NA) | NaN (NA) | NaN (NA) | 12 (5.9) | 12.8 (8.1) | 15.5 (9.8) | 8.2 (5.8) | 12.3 (9.1) | 15.7 (9.2) | NaN (NA) | NaN (NA) |
|  | 180 | 12.7 (7.3) | 9.4 (3.9) | 13.5 (7) | 11.3 (7) | 11.8 (7.7) | 10.1 (6.6) | 16.4 (7.9) | 15.1 (15.1) | 13 (0.9) | NaN (NA) | NaN (NA) | NaN (NA) | NaN (NA) | NaN (NA) | 19.9 (0.9) | 14.6 (8.6) | 11.4 (5.3) | 13.2 (4.1) | 17.7 (6.5) | 13.2 (9) | NaN (NA) | NaN (NA) |
| Bin 2 | | | | | | | | | | | | | | | | | | | | | | | |
|  | Age / Set Size | 4 | 5 | 6 | 7 | 8 | 9 | 10 | 11 | 12 | 13 | 14 | 15 | 16 | 17 | 18 | 19 | 20 | 21 | 22 | 23 | 24 | 25 |
| Feature | 60 | 13.7 (6.9) | 11.7 (8.2) | 18.3 (3.7) | 11.1 (7.6) | 14.4 (8.9) | 14 (8.6) | 15.2 (9.2) | 12.5 (7.5) | 12.8 (7.6) | 14.7 (9.7) | 9.3 (9.4) | 7.3 (9.8) | 8.5 (9.9) | -1.5 (0.7) | 13.8 (12) | 7.5 (NA) | 10.8 (11.9) | 20.9 (1.5) | 21.2 (1.4) | NaN (NA) | 21.7 (NA) | 20.9 (NA) |
|  | 100 | 8.4 (7.3) | 9.9 (7.8) | 10.2 (10.3) | 9.5 (7.1) | 15.4 (5.9) | 14.1 (8.1) | 15.2 (7.3) | 14.6 (10.2) | 6.6 (NA) | 24 (NA) | 20.9 (3.5) | 16.6 (5.9) | 11.9 (9.6) | NaN (NA) | 15.3 (8.9) | 16.5 (5.8) | 12.4 (5.9) | 14.2 (6.1) | 19.5 (7.3) | NaN (NA) | 10.4 (NA) | NaN (NA) |
|  | 140 | 5.8 (6) | 6.5 (6.5) | 10.2 (7) | 7.4 (7) | 8.2 (7.3) | 7.6 (5.3) | 11.8 (8.4) | 12.4 (9.4) | 8.3 (8.1) | 13.4 (9.6) | 13.6 (8.8) | 10.6 (8.4) | 10.9 (8.5) | 15.2 (10.5) | 14 (9.1) | 11 (11.2) | 8.6 (5.6) | 14.1 (8.9) | 16.6 (6.9) | NaN (NA) | 17 (10) | 16.1 (4.5) |
|  | 180 | 7.6 (4.8) | 8.6 (6) | 8.5 (7.4) | 7.2 (5.6) | 9.2 (5.6) | 10.4 (6.5) | 11.1 (6.1) | 6.5 (3.3) | 6.9 (5.9) | 9.8 (7.9) | 11.4 (6.7) | 10.3 (8.4) | 8.2 (6.5) | 13.3 (9) | 14.3 (7.2) | 4.4 (3.1) | 10.1 (6.4) | 11.9 (10) | 14.3 (10.1) | NaN (NA) | 5.7 (2.7) | 5.2 (7.5) |
| Conjunction | 60 | 8.1 (9.2) | 7.4 (7.3) | 16.6 (7.9) | 8.9 (8) | 10.2 (2.1) | 9.1 (9.6) | 10.6 (7.3) | 19.9 (3.6) | NaN (NA) | NaN (NA) | 22.5 (NA) | NaN (NA) | NaN (NA) | NaN (NA) | NaN (NA) | 15.4 (2.6) | -0.9 (NA) | NaN (NA) | 16.2 (NA) | 1.1 (NA) | NaN (NA) | NaN (NA) |
|  | 100 | 12.4 (8.1) | 8.7 (8.4) | 11.2 (8.5) | 6 (5.1) | 13.6 (8.7) | 4.8 (5.6) | 7.5 (10.4) | NaN (NA) | 12.9 (10) | 4.5 (4.5) | NaN (NA) | NaN (NA) | NaN (NA) | NaN (NA) | 3.8 (6.8) | 11.2 (9) | 7.9 (11.2) | 8.8 (9.2) | 8.4 (6.5) | 23.2 (NA) | NaN (NA) | NaN (NA) |
|  | 140 | 10.2 (7.6) | 10.4 (8.3) | 9 (8) | 6.9 (5.4) | 6.6 (5.8) | 6.2 (3.6) | 8.9 (7.8) | 2.3 (0.4) | 3.1 (2.6) | NaN (NA) | NaN (NA) | NaN (NA) | NaN (NA) | NaN (NA) | 2.5 (1.3) | 6.8 (6.7) | 9.1 (8.8) | 2.3 (2.4) | 8.6 (9.8) | 9.1 (9.1) | NaN (NA) | NaN (NA) |
|  | 180 | 8 (7.4) | 7 (6.3) | 4.8 (3.7) | 5 (2.5) | 5.8 (3.9) | 5 (3.2) | 6.7 (5.7) | 2.6 (2.6) | 4.1 (0.4) | NaN (NA) | NaN (NA) | NaN (NA) | NaN (NA) | NaN (NA) | 6.3 (2.9) | 7 (5.7) | 3 (1.1) | 11.3 (9.2) | 7.6 (2.1) | 3.2 (1.9) | NaN (NA) | NaN (NA) |

Table S7: Means (sd) of ρ_Θ_ by condition, age, and bin.

| Bin 1 | | | | | | | | | | | | | | | | | | | | | | | |
| --- | --- | --- | --- | --- | --- | --- | --- | --- | --- | --- | --- | --- | --- | --- | --- | --- | --- | --- | --- | --- | --- | --- | --- |
|  | Age / Set Size | 4 | 5 | 6 | 7 | 8 | 9 | 10 | 11 | 12 | 13 | 14 | 15 | 16 | 17 | 18 | 19 | 20 | 21 | 22 | 23 | 24 | 25 |
| Feature | 60 | 0 (0.3) | 0 (0.2) | 0 (0.1) | 0 (0.2) | 0 (0.2) | 0.1 (0.2) | -0.1 (0.1) | -0.1 (0.2) | -0.1 (0.3) | 0 (0.1) | 0 (0.1) | 0 (0.1) | -0.1 (0.1) | 0.2 (0.2) | 0 (0) | -0.1 (NA) | 0 (0.1) | -0.1 (0.1) | 0 (0) | NaN (NA) | 0.1 (NA) | 0 (NA) |
|  | 100 | 0 (0.3) | 0 (0.3) | 0 (0.2) | -0.1 (0.3) | 0 (0.3) | 0 (0.3) | -0.1 (0.2) | 0.1 (0.1) | 0.1 (NA) | 0.2 (NA) | -0.1 (0.2) | -0.2 (0.3) | -0.3 (0.3) | NaN (NA) | 0 (0.2) | -0.2 (0.3) | 0 (0.3) | 0 (0.1) | -0.1 (0.2) | NaN (NA) | -0.3 (NA) | NaN (NA) |
|  | 140 | 0.1 (0.4) | 0.2 (0.4) | 0.1 (0.3) | 0 (0.4) | 0 (0.4) | 0 (0.5) | -0.1 (0.3) | -0.1 (0.3) | -0.1 (0.3) | 0 (0.3) | 0 (0.3) | -0.1 (0.3) | 0 (0.3) | 0 (0.4) | -0.2 (0.2) | 0.1 (0.2) | -0.1 (0.2) | -0.1 (0.3) | -0.3 (0.4) | NaN (NA) | -0.1 (0) | -0.1 (0.1) |
|  | 180 | 0.1 (0.4) | 0 (0.6) | 0 (0.4) | 0 (0.5) | -0.2 (0.4) | -0.3 (0.4) | -0.2 (0.3) | -0.2 (0.3) | -0.5 (0.5) | -0.3 (0.3) | -0.2 (0.5) | -0.4 (0.4) | -0.1 (0.3) | -0.2 (0.2) | -0.2 (0.2) | -0.6 (0.4) | -0.1 (0.4) | -0.2 (0.3) | -0.2 (0.1) | NaN (NA) | -0.2 (0.1) | -0.1 (0.3) |
| Conjunction | 60 | -0.1 (0.2) | 0 (0.2) | 0 (0.1) | 0 (0) | 0.2 (0.2) | 0 (0.2) | 0.1 (0.2) | 0.1 (0.2) | NaN (NA) | NaN (NA) | 0 (NA) | NaN (NA) | NaN (NA) | NaN (NA) | NaN (NA) | -0.1 (0.2) | -0.1 (NA) | NaN (NA) | 0 (NA) | -0.2 (NA) | NaN (NA) | NaN (NA) |
|  | 100 | 0 (0.2) | 0.3 (0.4) | 0 (0.3) | 0 (0.3) | 0.1 (0.1) | 0 (0.2) | 0.1 (0.5) | NaN (NA) | 0.3 (0.1) | 0 (0.1) | NaN (NA) | NaN (NA) | NaN (NA) | NaN (NA) | 0.3 (0.4) | 0 (0.1) | 0.1 (0.1) | 0.2 (0.1) | 0 (0.1) | 0.6 (NA) | NaN (NA) | NaN (NA) |
|  | 140 | 0 (0.2) | 0.1 (0.4) | 0 (0.3) | 0.1 (0.4) | -0.1 (0.3) | 0.1 (0.2) | 0.2 (0.5) | -0.2 (0.3) | 0.2 (0.5) | NaN (NA) | NaN (NA) | NaN (NA) | NaN (NA) | NaN (NA) | 0.1 (1) | 0 (0.5) | 0.3 (0.3) | 0.3 (0.5) | 0 (0.4) | 0.2 (0.2) | NaN (NA) | NaN (NA) |
|  | 180 | 0.1 (0.3) | -0.1 (0.4) | 0.2 (0.4) | 0.1 (0.3) | -0.5 (0.5) | -0.1 (0.4) | -0.1 (0.3) | 0.2 (0.8) | 0.3 (0.2) | NaN (NA) | NaN (NA) | NaN (NA) | NaN (NA) | NaN (NA) | -0.2 (0.4) | 0.1 (0.3) | 0 (0.5) | -0.2 (0.2) | -0.2 (0.3) | 0 (0.4) | NaN (NA) | NaN (NA) |
| Bin 2 | | | | | | | | | | | | | | | | | | | | | | | |
|  | Age / Set Size | 4 | 5 | 6 | 7 | 8 | 9 | 10 | 11 | 12 | 13 | 14 | 15 | 16 | 17 | 18 | 19 | 20 | 21 | 22 | 23 | 24 | 25 |
| Feature | 60 | 0 (0.3) | 0.1 (0.3) | -0.1 (0.2) | 0 (0.3) | 0 (0.2) | -0.1 (0.2) | 0 (0.1) | 0 (0.2) | 0.1 (0.1) | 0.1 (0.1) | 0 (0.1) | 0 (0.1) | 0 (0.2) | -0.2 (0.3) | -0.1 (0.1) | 0 (NA) | -0.1 (0.2) | -0.1 (0.2) | 0 (0) | NaN (NA) | 0 (NA) | 0 (NA) |
|  | 100 | 0 (0.3) | -0.2 (0.3) | 0.1 (0.4) | 0.2 (0.4) | 0 (0.4) | 0 (0.2) | -0.2 (0.2) | 0 (0.2) | -0.1 (NA) | 0.1 (NA) | 0 (0.1) | 0 (0.3) | 0 (0.4) | NaN (NA) | 0.1 (0.1) | 0.1 (0.3) | 0.1 (0.1) | 0 (0.3) | -0.1 (0.2) | NaN (NA) | -0.3 (NA) | NaN (NA) |
|  | 140 | 0 (0.4) | 0.1 (0.5) | 0 (0.4) | 0.2 (0.6) | 0.1 (0.4) | 0 (0.5) | 0 (0.5) | 0.1 (0.3) | -0.1 (0.3) | 0 (0.3) | 0.1 (0.3) | 0 (0.4) | -0.1 (0.3) | -0.1 (0.3) | 0 (0.2) | 0 (0.3) | -0.1 (0.2) | 0.1 (0.3) | 0.1 (0.2) | NaN (NA) | 0.2 (0.3) | 0 (0.2) |
|  | 180 | 0 (0.4) | -0.1 (0.4) | 0.4 (0.5) | 0 (0.6) | 0 (0.5) | -0.1 (0.5) | 0 (0.4) | -0.1 (0.5) | 0.2 (0.5) | 0 (0.2) | -0.3 (0.3) | -0.1 (0.4) | 0.1 (0.3) | -0.1 (0.3) | 0 (0.4) | 0.2 (1.2) | 0.1 (0.3) | -0.2 (0.2) | -0.2 (0.4) | NaN (NA) | 0.1 (0.6) | 0 (0.3) |
| Conjunction | 60 | 0 (0.1) | 0 (0.3) | 0 (0.1) | 0 (0.2) | -0.4 (0) | 0.1 (0.2) | -0.1 (0.4) | 0 (0) | NaN (NA) | NaN (NA) | 0 (NA) | NaN (NA) | NaN (NA) | NaN (NA) | NaN (NA) | 0 (0.1) | -0.1 (NA) | NaN (NA) | 0 (NA) | -0.1 (NA) | NaN (NA) | NaN (NA) |
|  | 100 | 0 (0.2) | 0.2 (0.4) | 0.1 (0.4) | 0.1 (0.4) | 0.1 (0.1) | 0.2 (0.5) | 0.3 (0.6) | NaN (NA) | -0.3 (0.1) | -0.2 (0.2) | NaN (NA) | NaN (NA) | NaN (NA) | NaN (NA) | 0.2 (0.7) | 0.1 (0.1) | -0.2 (0.2) | 0 (0.1) | -0.1 (0.7) | 0 (NA) | NaN (NA) | NaN (NA) |
|  | 140 | 0 (0.3) | 0 (0.4) | 0 (0.3) | 0.1 (0.3) | 0.2 (0.4) | 0.4 (0.5) | 0 (0.4) | -0.2 (0) | 0.2 (0.2) | NaN (NA) | NaN (NA) | NaN (NA) | NaN (NA) | NaN (NA) | 0.4 (0.5) | 0 (0.4) | 0 (0.3) | 0.3 (0.6) | -0.1 (0.2) | -0.3 (0.4) | NaN (NA) | NaN (NA) |
|  | 180 | -0.1 (0.5) | 0.1 (0.4) | 0.2 (0.4) | 0.2 (0.5) | -0.2 (0.3) | -0.1 (0.4) | 0.1 (0.4) | 0.2 (0.3) | 0.5 (0.8) | NaN (NA) | NaN (NA) | NaN (NA) | NaN (NA) | NaN (NA) | -0.2 (0.1) | 0.1 (0.3) | 0.4 (0.1) | 0.5 (0.5) | 0.1 (0.5) | -0.1 (0.5) | NaN (NA) | NaN (NA) |

1. Actually, the IES is larger when the efficiency is lower, so negative correlations must be interpreted as positive correlations with efficiency. [↑](#footnote-ref-1)
